# Zhuangyang Bushen Pill Attenuates Renal Injury in Chronic Glomerulonephritis by Suppressing the MAPK Signaling Pathway

**DOI:** 10.3390/ph19050682

**Published:** 2026-04-27

**Authors:** Ying Xu, Lanlan Li, Nana Zhang, Yiming Luo, Li Song, Heng Luo

**Affiliations:** 1 Key Laboratory of Plant Resource Conservation and Germplasm Innovation in Mountainous Region (Ministry of Education), College of Life Sciences/Institute of Agro-Bioengineering, Guizhou University, Guiyang 550025, China; 2 State Key Laboratory of Discovery and Utilization of Functional Components in Traditional Chinese Medicine, School of Pharmaceutical Sciences, Guizhou Medical University, Natural Products Research Center of Guizhou Province, Guiyang 550014, China; 3 Zhaotong Fuming Business Services Co., Ltd., Zhaotong 657600, China

**Keywords:** Zhuangyang Bushen Pill, ethnomedicine, chronic glomerulonephritis, network pharmacology, WGCNA, EGFR, DUSP1, MAPK pathway

## Abstract

**Background/Objectives:** Chronic glomerulonephritis (CGN) is a progressive chronic kidney disease that can ultimately advance to end-stage renal disease (ESRD). Zhuangyang Bushen Pill (ZYBSW) is a traditional Chinese herbal formulation derived from the Yi ethnic medicine of Yunnan Province, and it has been widely employed in folk practice for the amelioration of chronic nephritis and renal dysfunction. This study was designed to evaluate the therapeutic efficacy of ZYBSW against CGN and to provide preliminary insights into its underlying mechanisms of action. **Methods:** The nephropathy model was induced in mice by tail vein injection of ADR (10 mg/kg). Renal function was evaluated by measuring relevant biochemical parameters, and renal histopathological alterations were examined using HE staining. Chemical constituents of ZYBSW were analyzed by LC-MS/MS. Its mechanisms of action were investigated using network pharmacology, WGCNA, molecular docking, multiplex immunofluorescence, and Western blotting. **Results:** ZYBSW significantly reduced ACR by 88.9%, SCr by 56.4%, and BUN by 30.4%, increased ALB by 32.4%, and alleviated renal histopathological damage (all *p* < 0.01). LC-MS/MS analysis identified 419 chemical constituents in ZYBSW. Network pharmacology, WGCNA, and molecular docking experiments identified EGFR and DUSP1 as potential targets, and indicated the MAPK pathway as a key pathway. Mechanistic studies revealed that ZYBSW significantly inhibited EGFR expression in renal tissue, enhanced DUSP1 expression, and reduced the phosphorylation levels of ERK, JNK, and p38. **Conclusions:** This study reveals ZYBSW can effectively alleviate CGN, with EGFR and DUSP1 as likely therapeutic targets, and its mechanism of action primarily involves regulating the MAPK signaling pathway.

## 1. Introduction

The global burden of chronic kidney disease (CKD) continues to escalate, affecting an estimated 850 million individuals worldwide and posing a significant public health challenge [1,2]. Chronic glomerulonephritis (CGN), characterized by diffuse or focal glomerular damage and clinical manifestations including proteinuria, hematuria, hypertension, and edema, represents one of the primary etiologies of CKD, accounting for approximately 20% of all cases. Without effective intervention, CGN may inexorably progress to end-stage renal disease (ESRD) [3,4]. Current therapeutic strategies for CGN, including glucocorticoids, immunosuppressants, and renin–angiotensin system inhibitors, primarily aim to suppress aberrant immune responses. However, prolonged administration of these agents is frequently associated with serious complications, such as opportunistic infections and metabolic disorders, underscoring the urgent need for adjunctive therapies that can effectively mitigate renal inflammation while preserving kidney function [5,6].

Traditional Chinese medicine (TCM), characterized by multi-component, multi-target therapeutic effects and favorable safety profiles, offers distinct advantages in the management of complex chronic diseases. Zhuangyang Bushen Pill (ZYBSW), a traditional medicinal compound originating from the Yi ethnic community in Zhaotong City, Yunnan Province, synthesizes the therapeutic principles of two classical prescriptions. The first, Bushen Pill, documented in the Yuan Dynasty text Danxi Xinfa, has been traditionally employed for consumptive diseases to tonify the kidneys, strengthen yang, fortify the spleen, and boost qi [7]. The second, Wuzi Yanzong Pill, derived from the Ming Dynasty medical text Shesheng Zongmiao Fang, primarily addresses kidney deficiency-related disorders [8]. Together, these formulas aim to comprehensively regulate renal yin, yang, essence, and qi, thereby restoring essential physiological functions including essence storage and water metabolism. ZYBSW comprises multiple herbal components with recognized nephroprotective properties, including *Eucommia ulmoides* Oliv., which reduces urinary protein excretion in diabetic nephropathy [9,10]; *Panax quinquefolius* L., which ameliorates cisplatin-induced nephrotoxicity through ROS-mediated MAPK/NF-κB signaling [11,12]; and *Euryale ferox* Salisb., which improves renal function through regulation of Keap1/Nrf2/HO-1 and AMPK/mTOR pathways [13]. Additional components such as *Astragalus mongholicus* Bunge and *Lycium chinense* Mill., recognized for their dual food and medicinal properties, exhibit anti-inflammatory, antioxidant, and CKD progression-delaying effects [14,15,16]. Although the aforementioned studies, based on single-herb analysis, preliminarily suggest that ZYBSW may exert its nephroprotective effects through the synergistic action of multiple components, the pharmacological basis and underlying molecular mechanisms of ZYBSW’s comprehensive intervention in CGN have not yet been fully explored. Specifically, the potential targets and core signaling pathways upon which its therapeutic effects rely have not yet been identified, nor have the intrinsic mechanisms by which the various chemical components in the compound regulate these potential targets through synergistic interactions been systematically elucidated.

This study first identified the chemical constituents of ZYBSW. Subsequently, a mouse model of CGN was established by a single tail vein injection of ADR to evaluate the effects of ZYBSW on renal function, inflammatory responses, and renal histopathological damage. Based on the scientific hypothesis that ZYBSW, as a multi-ingredient traditional Chinese medicine formula, may synergistically regulate the pathological progression of CGN through a “multi-component, multi-target, multi-pathway” mechanism, this study combined network pharmacology prediction and weighted gene co-expression network analysis (WGCNA) to screen for potential targets and signaling pathways. Network pharmacology can predict the potential targets and pathways of ZYBSW at the level of “drug-target-disease” interaction networks [17]; however, its predictive results typically rely on public databases and lack disease-specific transcriptomic expression information. In contrast, WGCNA can identify key co-expression modules highly correlated with the pathological phenotype of CGN by constructing gene co-expression networks, thereby revealing functional gene clusters that synergistically regulate disease onset and progression [18]. In this study, the former approach was used to construct a preliminary ”component-target” association network, while the latter identified key co-expression modules highly correlated with pathological features such as renal dysfunction and inflammatory responses, with the two approaches complementing each other; finally, molecular docking was used to predict the binding patterns of potential components and targets, and the screening results were experimentally validated using multiplex immunofluorescence staining and Western blot analysis. This study aims to systematically elucidate the material basis and molecular mechanisms underlying ZYBSW’s treatment of CGN, thereby providing a scientific basis for the development and clinical application of ZYBSW.

## 2. Results

### 2.1. ZYBSW Can Ameliorate Typical Symptoms and Renal Edema in CGN Mice

**Figure 1:**
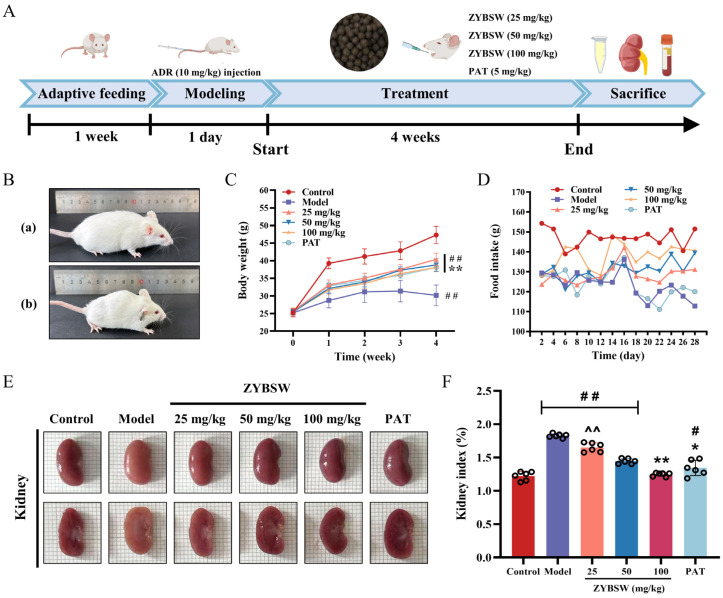
ZYBSW can ameliorate typical symptoms and renal edema in CGN mice. (**A**) Schematic diagram of animal model establishment. (**B**) Condition of control (**a**) and model (**b**) mice. (**C**) Body weight changes (*n* = 8). (**D**) Food intake statistics. (**E**) Macroscopic kidney surface images. Each small square is 1 mm on each side. (**F**) Kidney organ index statistics (*n* = 6). Data are expressed as mean ± SD. Compared with the control group, # *p* < 0.05; ## *p* < 0.01. Compared with the model group, * *p* < 0.05; ** *p* < 0.01. Compared with the PAT group, ^^ *p* < 0.01.

The therapeutic effectiveness of ZYBSW was assessed in a CGN mouse model, as outlined in Figure 1A. During the late modeling phase, mice in the model group (b) displayed prominent behavioral symptoms in comparison to the control group (a), such as lethargy, decreased spontaneous activity, hair loss, and kyphosis (Figure 1B). The results demonstrated that all treatment groups exhibited substantial improvements in body weight and food intake compared to the model group, indicating that ZYBSW intervention effectively delayed CGN-induced weight loss and reduced feeding patterns (Figure 1C,D). Examination of renal morphology (Figure 1E) showed that kidneys in the control group had a deep red color and a smooth, glossy surface, with clear differentiation between the cortex and medulla in cross-sections. Conversely, kidneys in the model group appeared pale red, fragile, and dull, with unclear cortical–medullary boundaries in cross-sections. Following ZYBSW intervention, there was a significant enhancement in kidney tissue color, gloss, and cortical demarcation in cross-sections. Further evaluation of kidney organ indices indicated notably elevated values in the model group, which substantially decreased after ZYBSW intervention, effectively reducing the severity of renal edema (Figure 1F). ZYBSW markedly improved typical symptoms and renal edema in CGN mice.

### 2.2. ZYBSW Enhances Renal Function, Decreases Inflammatory Factor Levels, and Regulates Blood Count-Related Parameters in CGN Mice

**Figure 2:**
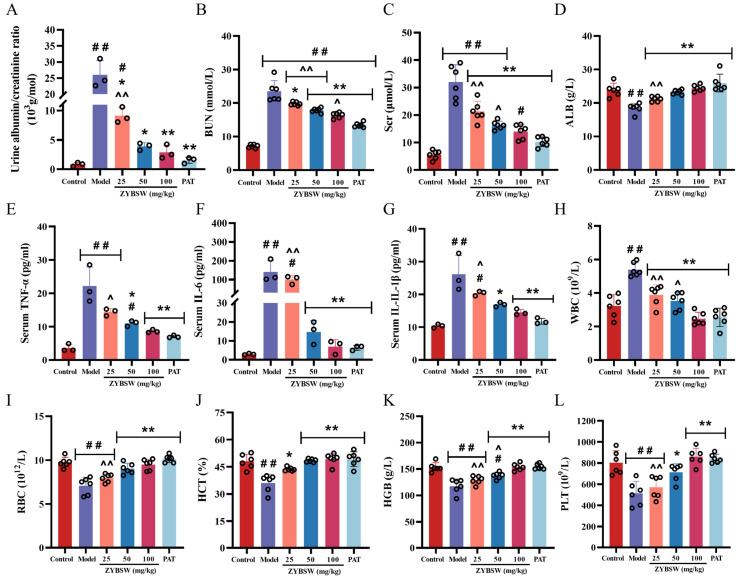
ZYBSW enhances renal function, decreases inflammatory factor levels, and regulates blood count-related parameters in CGN mice. (**A**) ACR (*n* = 3). (**B**) BUN (*n* = 6). (**C**) SCr (*n* = 6). (**D**) ALB (*n* = 6). (**E**) Serum TNF-α (*n* = 3). (**F**) Serum IL-6 (*n* = 3). (**G**) Serum IL-1β (*n* = 3). (**H**) White blood cell count (WBC) (*n* = 6). (**I**) Red blood cell count (RBC) (*n* = 6). (**J**) Hematocrit (HCT) (*n* = 6). (**K**) Hemoglobin (HGB) (*n* = 6). (**L**) Platelet count (PLT) (*n* = 6). Data are expressed as mean ± SD. Compared with the control group, # *p* < 0.05; ## *p* < 0.01. Compared with the model group, * *p* < 0.05; ** *p* < 0.01. Compared with the PAT group, ^ *p* < 0.05; ^^ *p* < 0.01.

Compared with the control group, mice in the model group exhibited significant renal impairment (*p* < 0.01), as evidenced by an increase in ACR from 0.83 ± 0.33 to 26.01 ± 4.46 × 10^3^ g/mol; an increase in Scr from 5.51 ± 1.64 to 32.05 ± 6.57 μmol/L; an increase in BUN from 7.11 ± 0.52 to 23.51 ± 3.18 mmol/L; and a significant decrease in ALB levels from 23.90 ± 2.00 to 18.45 ± 1.42 g/L. Following ZYBSW intervention, the aforementioned parameters improved significantly. Compared with the model group (*p* < 0.01), the high-dose ZYBSW group showed significantly reduced levels of ACR (2.90 ± 1.21 × 10^3^ g/mol), Scr (13.97 ± 2.62 μmol/L), and BUN (16.37 ± 0.83 mmol/L), while ALB levels increased to 24.43 ± 0.95 g/L, indicating that ZYBSW can effectively improve renal dysfunction in CGN mice (Figure 2A–D). Further analysis of inflammatory factors revealed that, compared with the control group, serum levels of IL-1β, IL-6, and TNF-α were significantly elevated in the model group (*p* < 0.01), reaching 26.14 ± 5.71, 140.47 ± 59.40, and 22.23 ± 5.63, respectively. This suggests that the CGN model successfully induced a systemic inflammatory response, whereas ZYBSW intervention significantly reduced the levels of these inflammatory factors (*p* < 0.01). In the high-dose group, IL-1β, IL-6, and TNF-α decreased to 14.54 ± 0.90, 6.99 ± 3.65, and 8.61 ± 0.46 (Figure 2E–G), respectively, indicating its anti-inflammatory effect. In addition, hematological analysis revealed that, compared with the control group, mice in the model group exhibited an approximately 40.3% increase in white blood cell (WBC) count (*p* < 0.01), while red blood cell (RBC), hemoglobin (HGB), hematocrit (HCT), and platelet (PLT) counts decreased by approximately 27.3%, 23.3%, 24.9%, and 36.0%, respectively (*p* < 0.01), which are characteristic of renal anemia and inflammation; following high-dose ZYBSW intervention, all of the above indicators improved significantly (*p* < 0.01): the white blood cell count decreased by approximately 54.3% compared to the model group, while the red blood cell count, hemoglobin, hematocrit, and platelet counts increased by approximately 33.9%, 30.7%, 36.7%, and 67.2% (Figure 2H–L), respectively, further confirming that ZYBSW can alleviate the inflammatory state and improve renal anemia in CGN mice.

### 2.3. ZYBSW Ameliorates Histopathological Alterations in CGN Mice and Inhibits the Expression of Inflammatory Cytokines

**Figure 3:**
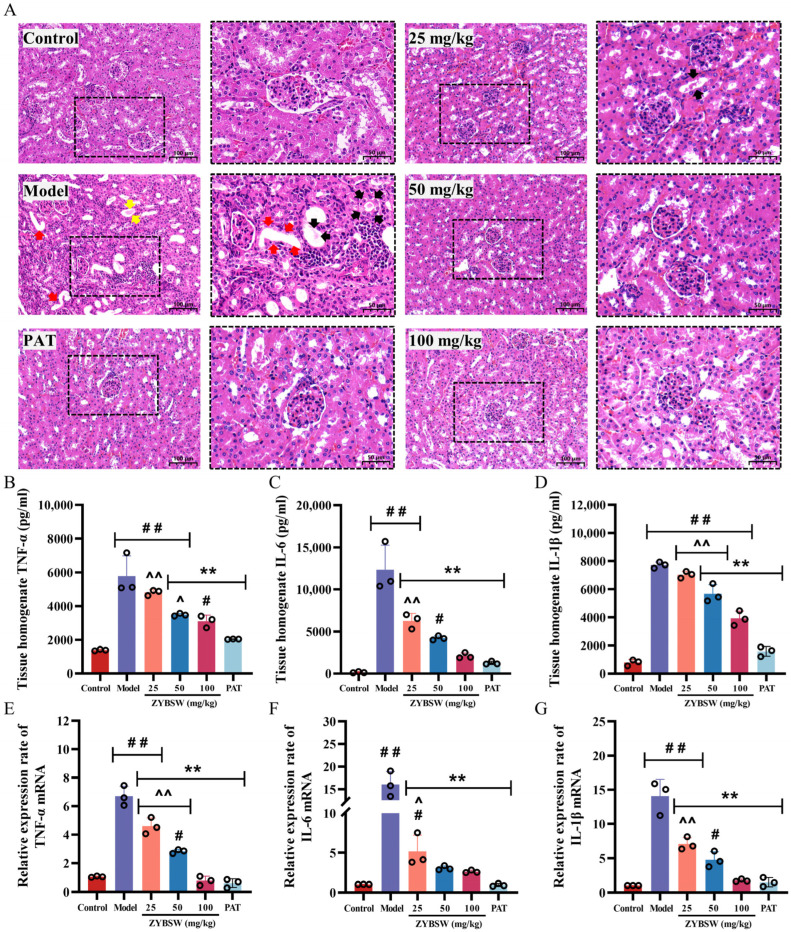
ZYBSW ameliorates histopathological alterations in CGN mice and inhibits the expression of inflammatory cytokines. (**A**) HE staining (*n* = 3; magnification 100×, scale bar = 100 μm; magnification 200×, scale bar = 50 μm). Yellow arrows indicate hyaline tubules. Black arrows indicate tubular dilatation. Red arrows indicate tubular lumens resembling thyroid follicles. (**B**–**D**) Tissue homogenate levels of IL-1β, IL-6, and TNF-α (*n* = 3). (**E**–**G**) Relative expression levels of IL-1β, IL-6, and TNF-α mRNA in kidney tissue (*n* = 3). Data are expressed as mean ± SD. Compared with the control group, # *p* < 0.05; ## *p* < 0.01. Compared with the model group, ** *p* < 0.01. Compared with the PAT group, ^ *p* < 0.05; ^^ *p* < 0.01.

Histopathological analysis indicated that the renal tissue structure in the control group was intact, with no significant abnormalities detected. In contrast, renal tissues from CGN mice displayed pronounced pathological changes, including dilated tubular lumens (black arrows) filled with homogeneously red-stained gelatinous casts resembling thyroid follicles (red arrows), as well as hyaline casts (yellow arrows). The renal interstitium exhibited extensive infiltration of inflammatory cells (Figure 3A). Following ZYBSW treatment, these pathological features were significantly reduced, accompanied by marked improvement in inflammatory infiltration. Consistent results were observed across multiple non-overlapping fields of view in each section, supporting the reproducibility of the qualitative findings. Additionally, levels of IL-6, IL-1β, and TNF-α in renal homogenates from the model group were significantly elevated (*p* < 0.01); following high-dose ZYBSW treatment, these levels decreased by 49.1%, 82.9%, and 46.2%, respectively, compared with the model group (*p* < 0.01) (Figure 3B–D). The mRNA expression levels of these inflammatory factors were also consistently upregulated (Figure 3E–G). In models, IL-1β mRNA, IL-6 mRNA, and TNF-α mRNA were 14.05 ± 2.47, 16.04 ± 2.85, and 6.70 ± 0.70, respectively, significantly higher than those in the control group (*p* < 0.01); in the high-dose ZYBSW group, the mRNA levels of these inflammatory factors decreased to 1.74 ± 0.20, 2.61 ± 0.18, and 0.77 ± 0.31, respectively, returning to levels close to those of the normal control group (*p* < 0.01). These findings suggest that ZYBSW mitigates renal pathological damage in CGN mice by suppressing the expression of inflammatory factors in renal tissue, thereby reducing local inflammatory responses.

### 2.4. Chemical Composition Identification and Network Pharmacology Analysis of ZYBSW

**Figure 4:**
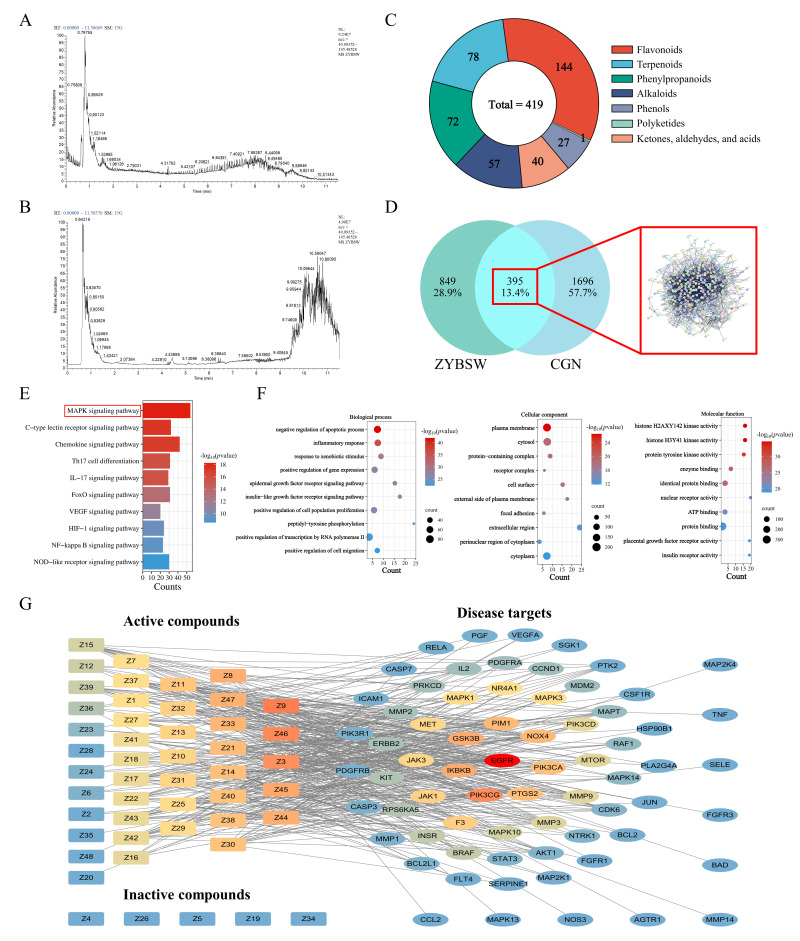
Chemical composition identification and network pharmacology analysis of ZYBSW. (**A**) Total ion current profile of ZYBSW in positive ion mode. (**B**) Total ion current profile of ZYBSW in negative ion mode. (**C**) Chemical composition classification and quantitative statistics of ZYBSW. (**D**) Venn diagram of ZYBSW drug targets and CGN disease targets. (**E**) KEGG pathway analysis. (**F**) GO pathway analysis (BP: biological process; CC: cellular component; MF: molecular function). (**G**) Active component–disease target interaction network diagram.

As shown in Figure 4A,B, comprehensive phytochemical analysis of the ZYBSW formulation was conducted under both positive and negative ion modes using a Thermo Vanquish (Thermo Fisher Scientific, Waltham, MA, USA) ultra-high-performance liquid chromatography system and an ACQUITY UPLC^®^ HSS T3 column (2.1 × 100 mm, 1.8 µm) (Waters, Milford, MA, USA). ZYBSW contained 419 components, with flavonoids being the most abundant group (144 compounds), followed by terpenoids (78 compounds) and phenylpropanoids (72 compounds). Alkaloids accounted for 57 compounds; ketones, aldehydes, and acids totaled 40; phenols comprised 27; and polyketones included only 1 (Figure 4C and Table S1). Network pharmacology methods predicted potential therapeutic targets of ZYBSW for CGN. A total of 2091 CGN-related disease targets were identified from three disease databases: Genecards, OMIM, and CTD. Intersection analysis between these disease targets and 1244 predicted drug targets from ZYBSW components identified 395 common targets (Figure 4D). Subsequent GO functional enrichment and KEGG pathway enrichment analyses were conducted on these 395 intersecting genes. KEGG enrichment results suggested that the intervention mechanism of ZYBSW in CGN may be closely linked to the “MAPK signaling pathway” (Figure 4E). GO enrichment analysis indicated that, in biological processes (BPs), the intersecting genes were significantly enriched in processes such as inflammatory response and regulation of cell proliferation. Cellular component (CC) analysis demonstrated that gene products were predominantly localized in the extracellular space, cell surface, and cytoplasm. In terms of molecular function (MF), gene products were primarily associated with protein tyrosine kinase activity, enzyme binding, and nucleic acid binding (Figure 4F). The interactions between active ingredients and intersecting targets were further analyzed using a protein–protein interaction (PPI) network, and the betweenness centrality (Betweenness) of each node was calculated. The Betweenness value reflects the importance of a target as a signal transduction bridge within the network; a higher value indicates a greater potential for mediating the synergistic effects of multi-ingredient drugs. Therefore, using the Betweenness value as the screening threshold, five major active ingredients were ultimately identified (Table 1), along with the potential target gene EGFR, which had the highest Betweenness value (Figure 4G).

### 2.5. WGCNA Screening and Experimental Validation Were Conducted to Identify Potential Targets for ZYBSW Treatment in CGN

**Figure 5:**
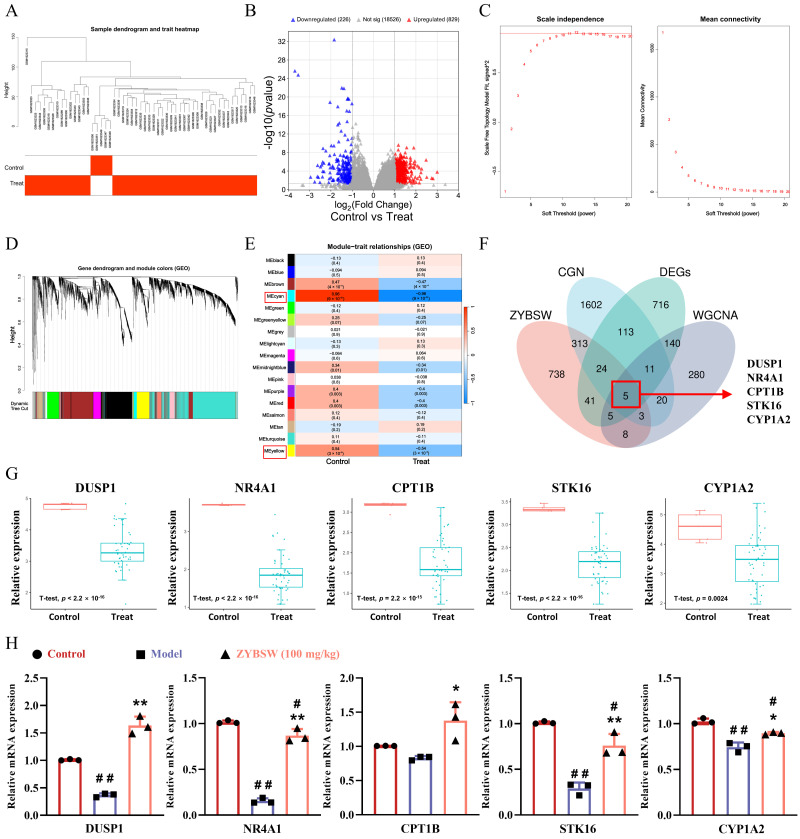
WGCNA screening and experimental validation were conducted to identify potential targets for ZYBSW treatment in CGN. (**A**) Sample clustering heatmap. (**B**) Volcano plot of differentially expressed genes. Red indicates significantly upregulated genes; blue indicates significantly downregulated genes. (**C**) R^2^ values corresponding to different soft thresholds (left) and gene adjacency coefficients corresponding to different soft thresholds (right). (**D**) Gene clustering diagram after merging similar gene modules. (**E**) Heatmap of module–phenotype correlations, showing correlation coefficients between gene modules and disease/normal phenotypes. (**F**) Venn diagram illustrating the intersection of CGN disease targets, ZYBSW drug prediction targets, GSE66494 differentially expressed genes, and WGCNA key module genes. (**G**) Single-gene differential expression analysis of intersecting genes *DUSP1*, *NR4A1*, *CPT1B*, *STK16*, and *CYP1A2* in the GSE66494 dataset. (**H**) RT-qPCR validation of relative mRNA expression levels for intersecting genes DUSP1, NR4A1, CPT1B, STK16, and CYP1A2 in mouse kidney tissue (*n* = 3). Data are expressed as mean ± SD. Compared with the control group, # *p* < 0.05; ## *p* < 0.01. Compared with the model group, * *p* < 0.05; ** *p* < 0.01.

Hierarchical clustering of the GSE66494 dataset (GEO database) generated a sample dendrogram (Figure 5A) and identified 1055 differentially expressed genes, comprising 829 upregulated and 226 downregulated genes (Figure 5B). Subsequently, weighted gene co-expression network analysis (WGCNA) was performed. A soft threshold of β = 10 was chosen to construct the adjacency matrix (Figure 5C). Hierarchical clustering based on gene adjacency coefficients resulted in a gene clustering dendrogram (Figure 5D). Further analysis of gene module correlations with CGN disease status identified 17 co-expression modules (Figure 5E). According to the standard in biomedical correlation analysis, where |r| > 0.5 indicates a moderate correlation and serves as the commonly used cutoff value in WGCNA, the MEcyan module (r = 0.96) and the MEyellow module (r = 0.54) met this criterion and had a corrected *p*-value < 0.01; therefore, they were identified as key modules. To identify potential therapeutic targets for ZYBSW in CGN, a multivariate Venn analysis was conducted, incorporating CGN disease targets, predicted ZYBSW drug targets, differentially expressed genes from the GSE66494 dataset, and genes from two key modules. The intersection of these four approaches can effectively reduce the false positive rate in a single dataset and identify genes that are differentially expressed in the disease and are closely associated with drug and co-expression networks. The analysis identified *DUSP1*, *NR4A1*, *STK16*, *CPT1B*, and *CYP1A2* as potential therapeutic targets for ZYBSW in CGN (Figure 5F). A single-gene differential expression analysis of these five genes within the GSE66494 dataset demonstrated consistent downregulation in the CGN model (Figure 5G). Further validation at the animal level was performed using RT-qPCR to assess the relative mRNA expression levels of these genes in kidney tissues from the control, model, and ZYBSW (100 mg/kg) groups. Consistent with the single-gene differential expression analysis, the expression of all five genes was significantly downregulated in the model group, whereas ZYBSW intervention effectively reversed this trend (Figure 5H). These findings suggest that these genes may play a role in the therapeutic mechanism of ZYBSW for CGN.

### 2.6. Molecular Docking Validation of ZYBSW Active Compounds with Potential Targets

**Figure 6:**
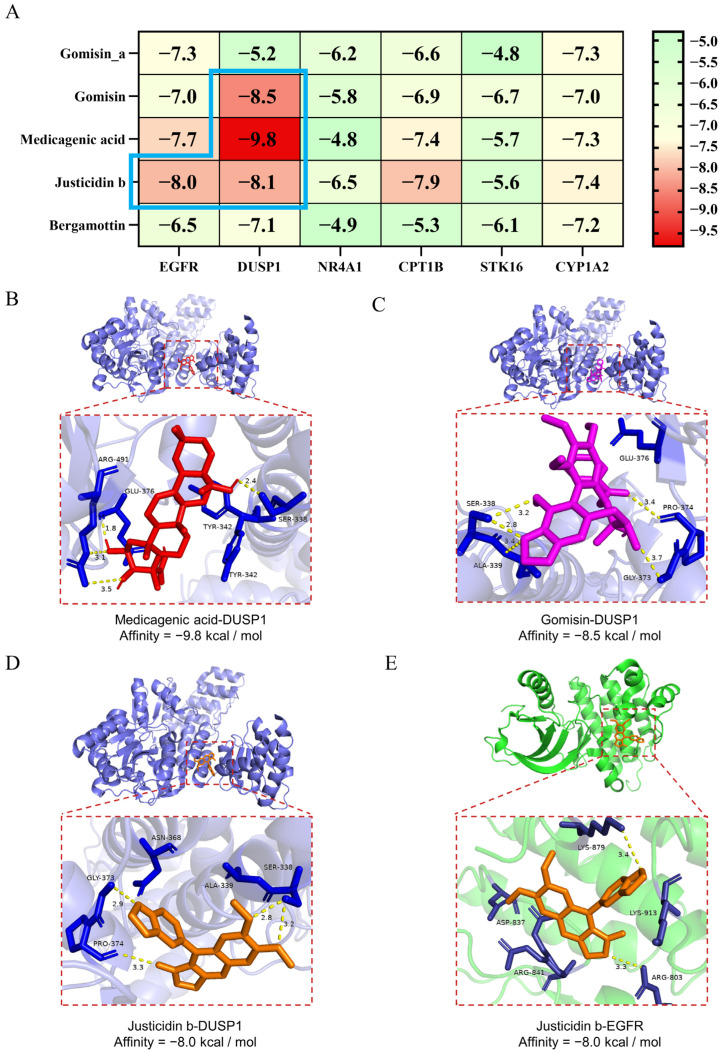
Molecular docking validation of ZYBSW active compounds with potential targets. (**A**) Heatmap of docking binding energies between 5 active compounds and 6 potential target molecules. (**B**) Medicagenic acid with DUSP1 (binding energy: −9.8 kcal/mol); (**C**) Gomisin with DUSP1 (binding energy: −8.5 kcal/mol); (**D**) Justicidin b with DUSP1 (binding energy: −8.0 kcal/mol); (**E**) Justicidin b with EGFR (binding energy: −8.0 kcal/mol).

This study examined the interactions between five selected active compounds (Gomisin, Medicagenic acid, Gomisin A, Bergamottin, and Justicidin B) and target proteins, including EGFR, DUSP1, NR4A1, CPT1B, STK16, and CYP1A2. Binding energies were represented in a heatmap (Figure 6A), where lower values indicate more stable ligand–receptor interactions. The docking results suggested potential binding affinity between the active compounds and target proteins. The most favorable binding energy pairs were observed with Medicagenic acid and DUSP1 (−9.8 kcal/mol, Figure 6B), Gomisin and DUSP1 (−8.5 kcal/mol, Figure 6C), Justicidin b and DUSP1 (−8.0 kcal/mol, Figure 6D), and Justicidin b and EGFR (−8.0 kcal/mol, Figure 6E). In conclusion, based on these supportive docking results, Medicagenic acid, Gomisin, and Justicidin b are suggested as potential active components in ZYBSW, potentially modulating the MAPK signaling pathway by targeting EGFR and DUSP1. It should be noted that these docking results provide computational support for subsequent experimental validation rather than definitive confirmation. Subsequent research will concentrate on experimentally validating these potential targets and the associated MAPK signaling pathway to further elucidate ZYBSW’s molecular mechanism in treating CGN.

### 2.7. Investigation of the Effects of ZYBSW on EGFR and DUSP1 Expression and the MAPK Signaling Pathway in Kidney Tissues of CGN Mice

**Figure 7:**
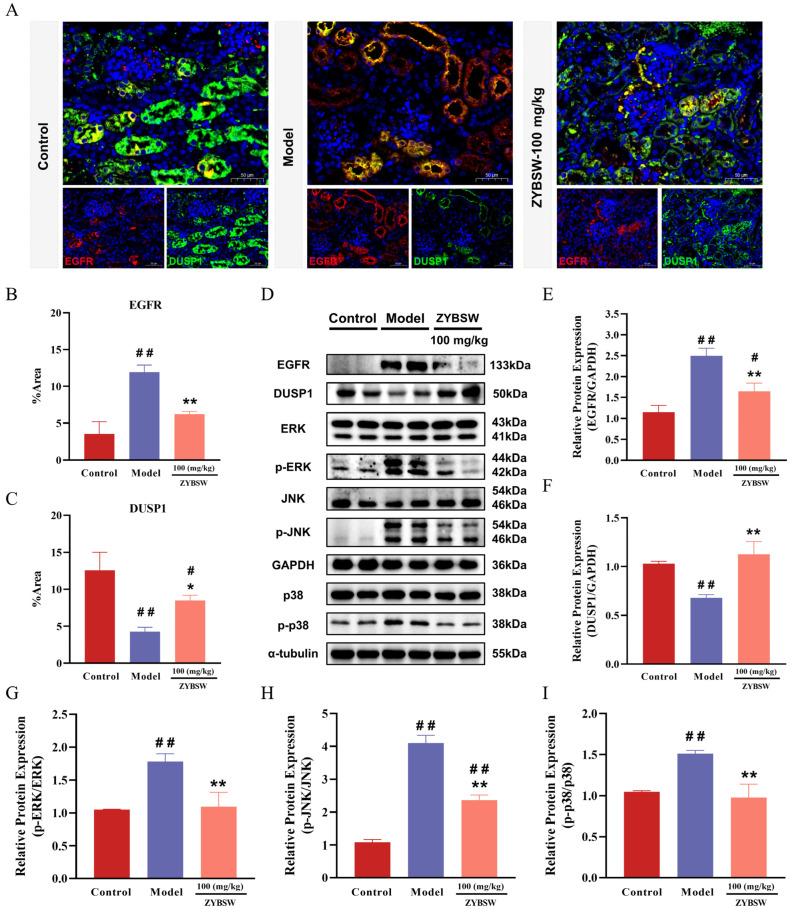
Effects of ZYBSW on EGFR and DUSP1 expression and the MAPK signaling pathway in CGN mice kidney tissue were investigated. (**A**) Representative images of immunofluorescence staining for EGFR (red), DUSP1 (green), and DAPI (blue) in kidneys from each group of mice (magnification 50×, scale bar = 50 μm). ImageJ 1.8.0 software was used to quantify the (**B**) EGFR-positive area percentage and (**C**) DUSP1-positive area percentage. (**D**) Western blot detection of EGFR, DUSP1, and MAPK pathway-related protein expression levels in kidney tissue, with α-tubulin and GAPDH as internal controls. (**E**) Quantitative analysis of relative EGFR protein expression levels. (**F**) Quantitative analysis of relative DUSP1 protein expression. (**G**) Quantitative analysis of the p-ERK/ERK ratio. (**H**) Quantitative analysis of the p-JNK/JNK ratio. (**I**) Quantitative analysis of the p-p38/p38 ratio. Data are expressed as mean ± SD, *n* = 3. Compared with the control group, # *p* < 0.05; ## *p* < 0.01. Compared with the model group, * *p* < 0.05; ** *p* < 0.01.

mIHC results (Figure 7A) indicated that, in comparison to the control group, EGFR fluorescence signals were significantly elevated in the renal tubular regions of model group mice, while DUSP1 expression was notably decreased. Following ZYBSW intervention, these changes were significantly reversed: EGFR expression in the renal tubular region was diminished, whereas DUSP1 expression increased. Quantitative analysis further corroborated that, relative to the model group, the ZYBSW group exhibited a significantly reduced percentage of EGFR-positive area and a significantly elevated level of DUSP1-positive expression (Figure 7B,C). Western blot results confirmed these findings at the protein level (Figure 7D). Compared to the control group, EGFR protein expression was significantly upregulated, and DUSP1 protein expression was significantly downregulated in the kidney tissues of the model group. Conversely, ZYBSW intervention significantly downregulated EGFR expression and upregulated DUSP1 expression compared to the model group (Figure 7E,F). Further analysis investigated the phosphorylation levels of key proteins within the MAPK pathway. The results revealed no significant differences in total ERK, total JNK, or total p38 expression across the groups. In comparison to the control group, the model group exhibited significantly elevated levels of p-ERK, p-JNK, and p-p38 proteins. Quantitative analysis demonstrated that the ratios of p-ERK/ERK, p-JNK/JNK, and p-p38/p38 were significantly higher in the model group than in the control group, indicating excessive activation of the MAPK signaling pathway in the renal tissues of CGN model mice. In contrast, ZYBSW intervention significantly reduced the levels of p-ERK, p-JNK, and p-p38 proteins in the model group, leading to a marked decrease in the ratios of p-ERK/ERK, p-JNK/JNK, and p-p38/p38 (Figure 7G–I). These findings suggest that ZYBSW inhibits EGFR expression, enhances DUSP1 expression, and significantly downregulates the phosphorylation levels of key MAPK pathway proteins ERK, JNK, and p38, thereby attenuating the excessive activation of the MAPK signaling pathway. This regulatory mechanism may provide a crucial molecular basis for ZYBSW’s alleviation of renal tubular injury in CGN mice.

## 3. Discussion

Chronic glomerulonephritis (CGN) is an immune-mediated disorder characterized by glomerular inflammation, interstitial inflammatory infiltration, and progressive fibrosis, ultimately leading to nephron loss [19,20]. Current immunosuppressive therapies demonstrate limited long-term efficacy and significant adverse effects [21,22]. Traditional Chinese medicine formulations, with their multi-component and multi-target characteristics, offer distinct advantages in CGN treatment [23,24]. This study investigated the therapeutic components and mechanisms of Zhuangyang Bushen Pill (ZYBSW) through phytochemical analysis, network pharmacology, WGCNA, and experimental validation.

LC-MS/MS analysis identified 419 compounds in ZYBSW, predominantly flavonoids, terpenoids, phenylpropanoids, and alkaloids—all recognized for their anti-inflammatory, antioxidant, and nephroprotective properties [25,26,27,28,29,30]. Notably, these components constitute the material basis for the therapeutic efficacy of ZYBSW in treating CGN. In vivo experiments demonstrated that ZYBSW significantly reduced SCr and BUN levels, increased ALB content, and ameliorated renal pathological damage including tubular dilatation, hyaline cast formation, and inflammatory cell infiltration. Scr, BUN, and urine protein are classic markers of glomerular filtration and kidney injury, while ALB reflects protein loss in kidney disease [31,32]. Based on the above phenotypic results, it is inferred that ZYBSW effectively alleviates CGN symptoms and improves renal function, consistent with its traditional use in treating chronic nephritis and renal dysfunction.

Integrating network pharmacology with WGCNA analysis identified EGFR and DUSP1 as core therapeutic targets, with KEGG enrichment highlighting the MAPK signaling pathway—a critical intracellular cascade regulating inflammatory responses, cell proliferation, and apoptosis [33,34,35,36]. Aberrant MAPK activation in kidney diseases correlates closely with tubular injury, podocyte apoptosis, and interstitial fibrosis [37,38,39,40]. Many studies have utilized molecular docking to predict interactions between plant compounds and target proteins [41,42,43]. Building upon these established computational approaches, we performed molecular docking to investigate whether compounds from ZYBSW could directly interact with EGFR and DUSP1, two key proteins associated with nephroprotection and anti-inflammation. Molecular docking provided evidence that ZYBSW-derived compounds—Medicagenic acid, Gomisin, and Justicidin b—exhibited strong binding affinities (binding energies < −8.0 kcal/mol) to EGFR and DUSP1. Notably, Medicagenic acid demonstrated particularly high affinity for DUSP1 (−9.8 kcal/mol), suggesting potential direct interaction to stabilize DUSP1 or enhance its expression. These compounds possess documented anti-inflammatory and nephroprotective activities [44,45,46], supporting their candidacy as active constituents responsible for ZYBSW’s therapeutic effects. Nevertheless, molecular docking is a computational simulation that does not account for pharmacokinetics or in vivo binding; thus, these binding predictions require experimental validation.

Experimental validation confirmed that CGN model mice exhibited significantly upregulated EGFR expression and downregulated DUSP1 expression in renal tissues. EGFR mediates kidney development and tissue repair [47], while DUSP1 negatively regulates MAPK signaling through dephosphorylation, suppressing cellular stress responses and inflammation [48,49]. ZYBSW treatment concurrently suppressed EGFR expression and promoted DUSP1 expression. Furthermore, model mice showed elevated phosphorylation levels of ERK, JNK, and p38 without changes in total protein levels, indicating MAPK pathway overactivation. Following ZYBSW intervention, MAPK phosphorylation significantly decreased alongside normalized EGFR and DUSP1 expression, suggesting that ZYBSW-mediated MAPK inhibition occurs through coordinated regulation of these upstream modulators. These mechanistic findings demonstrated that ZYBSW exerts its effects through regulation of the MAPK signaling pathway involving EGFR and DUSP1. However, the observed associations among EGFR, DUSP1, and MAPK phosphorylation are correlational; whether DUSP1 upregulation is a cause or consequence of MAPK inhibition remains unresolved, and whether ZYBSW directly targets EGFR/DUSP1 or acts through intermediate molecules is unknown.

Based on experimental results from phytochemical analysis, network pharmacology combined with WGCNA screening, molecular docking simulations, and in vivo validation, this study systematically investigated the material basis and molecular mechanism of ZYBSW in treating CGN following the “component–efficacy–mechanism” research framework. Phytochemical analysis revealed that ZYBSW contains 419 compounds, including Medicagenic acid, Gomisin, and Justicidin b, many of which have been reported to possess anti-inflammatory and nephroprotective properties. Combined network pharmacology and WGCNA analysis identified EGFR and DUSP1 as potential targets, and KEGG enrichment indicated the MAPK signaling pathway as a key pathway. Molecular docking predicted that the binding energies of the three aforementioned compounds with EGFR and DUSP1 were all below –8.0 kcal/mol, suggesting potential direct interactions. In vivo experiments confirmed that ZYBSW simultaneously downregulated EGFR expression, upregulated DUSP1 expression, and significantly reduced the levels of p-ERK, p-JNK, and p-p38, while also improving renal function and renal histopathological damage. In summary, this study found that the active components of ZYBSW (e.g., Medicagenic acid, Gomisin, Justicidin b) may synergistically inhibit excessive activation of the MAPK signaling pathway by targeting the downregulation of EGFR and upregulation of DUSP1, thereby alleviating renal tubular injury and inflammatory responses, and improving renal function and systemic symptoms. These findings provide a scientific basis for the clinical application of ZYBSW and offer a novel insight into the treatment strategy for CGN using traditional Chinese medicine formulas.

This study still has certain limitations. The regulatory relationships among EGFR, DUSP1, and the MAPK pathway are currently based primarily on expression correlation analyses; however, the causal relationships among these three factors have not yet been fully elucidated. For example, the upstream–downstream relationship between EGFR and DUSP1 in the activation of the MAPK pathway, as well as the direct and indirect effects of ZYBSW on these two targets, still requires further validation through intervention experiments such as gene silencing and overexpression. Molecular docking provides computational evidence for potential binding; however, its computational approach has inherent limitations. Moreover, molecular docking results are derived from computer simulations that neglect protein and ligand flexibility, approximate solvation and entropy effects, and cannot account for pharmacokinetic properties. The in vitro and in vivo activities of the relevant bioactive compounds, as well as their direct binding to target proteins, require experimental confirmation.

## 4. Materials and Methods

### 4.1. Chemicals and Reagents

ZYBSW is derived from an enhanced traditional Chinese herbal formula originating in Yunnan and was supplied by Zhaotong Fuming Business Service Co., Ltd. (Zhaotong, Yunnan Province, China); Adriamycin (A864033) was acquired from Shanghai Macklin Biochemical Technology (Shanghai, China). Prednisone acetate tablets (PAT) (LA24208, specification: 5 mg) were sourced from Zhejiang Xianju Pharmaceutical Co., Ltd. (Taizhou, Zhejiang Province, China); Urea nitrogen (C013-2-1) and creatinine (C011-2-1) assay kits were obtained from Nanjing Jiancheng Biotechnology Research Institute Co., Ltd. (Nanjing, Jiangsu Province, China); Albumin (JL-T0745) and mouse ELISA kits for IL-1β (JL13662), IL-6 (JL20268), and TNF-α (JL10484) were procured from Shanghai Jianglai Biotechnology Co., Ltd. (Shanghai, China); The FastKing cDNA First Strand Synthesis Kit (Genome-Free) (B0507A) was purchased from Beijing Tiangen Biochemical Technology Co., Ltd. (Beijing, China); SYBR dye (11190ES08) was obtained from Shanghai Yisheng Biotechnology Co., Ltd. (Shanghai, China); The BCA protein assay kit (BL521A) was acquired from Biosharp (Beijing, China). EGFR (EP38Y) antibody was purchased from Abcam Shanghai Trading Co., Ltd. (Shanghai, China); Antibodies for DUSP1 (JJ0930), ERK (SA43-03), p-ERK (SZ2-4), JNK (SA43-06), p-JNK (ST500), p38 (SR43-04), p-p38 (JE77-37), and GAPDH (ET1601-4) were sourced from Hangzhou Hua’an Biotechnology Co., Ltd. (Hangzhou, Zhejiang Province, China); α-tubulin (GB15201) was obtained from Wuhan Savir Biotechnology Co., Ltd. (Wuhan, Hubei Province, China); Other reagents and solvents were of analytical grade or commercially available and were used without further purification.

### 4.2. Preparation of ZYBSW

ZYBSW comprises a blend of Chinese herbs, including *Schisandra chinensis* (Turcz.) Baill. (10–20 parts), *Epimedium brevicornu* Maxim. (15–30 parts), *Allium tuberosum* Rottler ex Spreng. (20–40 parts), *Cuscuta chinensis* Lam. (15–30 parts), *Lycium chinense* Mill. (15–20 parts), *Ziziphus jujuba* Mill. (20–40 parts), *Cistanche deserticola* Ma. (15–30 parts), *Panax quinquefolius* L. (15–30 parts), *Astragalus mongholicus* Bunge (10–20 parts), *Gynochthodes officinalis* (F.C.How) Razafim. & B.Bremer (15–30 parts), *Cervus nippon* Temminck (10–20 parts), *Eucommia ulmoides* Oliv. (10–20 parts), *Raphanus raphanistrum* subsp. *sativus* (L.) Domin (10–20 parts), *Rosa laevigata* Michx. (10–20 parts), *Polygonatum sibiricum* Redouté (10–20 parts), *Morus nigra* L. (20–40 parts), *Gastrodia elata* Blume (15–30 parts), *Coix lacryma-jobi* var. *ma-yuen* (Rom.Caill.) Stapf (20–40 parts), *Dioscorea polystachya* Turcz. (10–20 parts), and *Euryale ferox* Salisb. (10–20 parts). The ZYBSW mixture was ground into a powder and sieved through a 60-mesh screen. Three separate batches were prepared, each starting with 200 g of powder. Distilled water was added at a solid-to-liquid ratio of 1:10 (*w*/*v*), and the mixture was soaked at room temperature for 24 h; then it was heated in a 65 °C water bath for 60 min, followed by ultrasonic treatment for 30 min (300 W, 40 kHz, SB-5200DT); the mixture was filtered under vacuum and the filtrate was collected. The above extraction steps were repeated three times, and all filtrates were combined. The combined filtrate was concentrated using rotary evaporation at 55 °C until a thick paste was formed. After pre-freezing at −20 °C for 24 h, the mixture was placed in a vacuum freeze dryer (OLB-FD10P) and freeze-dried for 48 h under a vacuum of <1 Pa and a cold trap temperature of −55 °C to obtain the freeze-dried powder [50]. The freeze-dried powder was transferred to sterile vials and stored under tight-sealed conditions, with a yield of 11.2 ± 1.1%.

### 4.3. Animal Studies

Healthy adult male Kunming mice (5 weeks old, weighing 22–23 g) were procured from Guizhou Jiuxi Biotechnology Co., Ltd. (Guiyang, Guizhou Province, China) and accommodated in a specific pathogen-free facility with isolated ventilated cage (IVC) barrier conditions. The environmental conditions were controlled at (22 ± 2)°C, with humidity maintained at (50 ± 10)%, and a standard light–dark cycle was established. The mice were provided ad libitum access to sterile chow and water. This animal research was ethically reviewed and approved by the Guizhou University Subcommittee of Experimental Animal Ethics (EAE-GZU-2025-E071).

After one week of adaptive feeding, 60 mice were randomly divided into six groups (*n* = 10): control, model, PAT (5 mg/kg), and ZYBSW low-dose (25 mg/kg), medium-dose (50 mg/kg), and high-dose (100 mg/kg) groups. The experimental personnel who performed drug administration, biochemical measurements, and histopathological evaluations were blinded to the group assignments. Unblinding occurred only after the completion of all data collection. The dosage of ZYBSW was determined based on the results of preliminary experiments conducted by our research group, combined with clinical equivalence calculations for traditional medications [51]. The ADR-induced nephropathy model is currently one of the most widely used rodent models for studying human CKD. It induces pathological changes highly similar to those seen in human nephropathy, including proteinuria, podocyte damage, glomerulosclerosis, and tubulointerstitial fibrosis [52]. The Kunming mouse is a closed-colony strain widely used in China and has been successfully employed to establish this model [53,54]. With the exception of the control group, which received an equivalent volume of saline, the CGN mouse model was induced in all other groups via a single tail vein injection of ADR (10 mg/kg) [55,56]. Gastrointestinal administration of the drug commenced the day after modeling and was conducted once daily for four consecutive weeks. Both the control and model groups received an equivalent volume of physiological saline. Throughout the experiment, the general condition of the mice, including behavior, feeding habits, coat condition, and activity levels, was observed and recorded, along with weekly body weight changes. After the final drug administration, urine samples were collected from each group of mice using metabolic cages. Subsequently, blood and kidney tissue samples were obtained for further analysis.

### 4.4. Biochemical Index Determination

Urine samples collected were centrifuged at 4 °C at 3500 rpm for 10 min to determine urinary albumin and creatinine levels in mice using ALB and Cr kits according to the manufacturers’ protocols [57,58]. The results were expressed as the albumin-to-creatinine ratio (ACR, 10^3^ g/mol). An automated biochemical analyzer was utilized to evaluate blood chemistry parameters. Blood obtained from mice via eyeball puncture was centrifuged at 3500 rpm for 15 min at 4 °C. Subsequently, serum creatinine (Scr), blood urea nitrogen (BUN), and serum albumin (ALB) levels were analyzed following the manufacturer’s instructions [59,60].

### 4.5. ELISA

Serum and supernatants from kidney tissue homogenates were collected for analysis. The levels of the inflammatory cytokines IL-1β, IL-6, and TNF-α were quantified using mouse IL-1β, IL-6, and TNF-α ELISA kits, in accordance with the manufacturers’ protocols [61].

### 4.6. Histopathological Examination

Three kidneys from each group were randomly selected and fixed in 4% paraformaldehyde. Subsequently, the kidneys were embedded in paraffin, sectioned, and stained with hematoxylin and eosin (HE). The stained sections were scanned using a Panoramic SCAN II scanner (3DHISTECH Ltd., Budapest, Hungary).

### 4.7. Multiplex Immunohistochemistry (mIHC)

Following dewaxing of kidney tissue sections, antigen retrieval, endogenous enzyme inhibition, and serum blocking were conducted. Subsequently, primary antibodies targeting EGFR (1:200) and DUSP1 (1:500) were administered and left to incubate overnight at 4 °C. The sections underwent incubation with secondary antibodies, and were then treated with TSA reagent and dehydrated. Lastly, DAPI was used to stain the nuclei, and an anti-fade mounting medium was applied before sealing with coverslips. The stained sections were then scanned using a fluorescence scanner (PANNORAMIC MIDI, 3DHISTECH Ltd., Budapest, Hungary).

### 4.8. Network Pharmacology

Targets associated with CGN were identified using the GeneCards (https://www.genecards.org/, (accessed on 20 April 2025)), OMIM (https://omim.org/, (accessed on 20 April 2025)), and CTD (https://ctdbase.org/, (accessed on 1 August 2025)) databases. After merging the targets obtained from each database and removing duplicates, these targets were designated as CGN-associated targets. Compound SMILES were retrieved from PubChem (https://pubchem.ncbi.nlm.nih.gov/) and compound toxicity analysis and target prediction were conducted utilizing SwissADME and SwissTargetPrediction (https://swisstargetprediction.ch/, (accessed on 4 August 2025)). Among them, SwissADME was used to screen for components with high GI absorption and at least two “Yes” criteria, while SwissTargetPrediction retained targets with a probability value greater than 0. The intersecting genes were imported into the STRING database (http://string-db.org), the species was set to “Homo sapiens,” and a PPI network was constructed using a threshold of a minimum interaction confidence score of ≥0.7. Gene Ontology (GO) analysis and KEGG analysis were performed via the DAVID database (https://davidbioinformatics.nih.gov/, (accessed on 5 August 2025)), using a threshold of *p* < 0.05. The data were visualized using the MicroBioinformatics platform (http://www.com/, (accessed on 5 August 2025)). Finally, Cytoscape 3.10.3 software was employed to visualize the data, construct protein–protein interaction (PPI) networks, screen active compounds, and establish an “Active compound-Disease targets” network.

### 4.9. Weighted Gene Co-Expression Network Analysis (WGCNA)

The GSE66494 dataset, obtained from the GEO database (https://www.ncbi.nlm.nih.gov/geo/, (accessed on 9 February 2026)), was utilized to construct a gene co-expression network using specific R packages (Version: 4.5.1). Differential expression analysis was performed to identify differentially expressed genes using the screening criteria of |log2FC| ≥ 1 and *p* < 0.05, and a volcano plot was generated to visualize these findings. Outlier samples were detected and subsequently removed through sample clustering analysis, resulting in a sample clustering dendrogram. The optimal soft threshold β was calculated by selecting a value with a network fit index R^2^ > 0.8 and a relatively high average connectivity, after which the correlation coefficient matrix was converted into a topological overlap matrix (TOM) to construct a gene clustering dendrogram and a gene module clustering dendrogram using a cut height of 0.25. Hierarchical clustering algorithms were employed to create color-coded gene modules. Correlations between module features and sample information were assessed, with a threshold of |r| > 0.5 set for statistical significance, allowing for the selection of modules exhibiting the most significant positive or negative correlations as key module genes. Key genes were identified by integrating network pharmacology results and were further analyzed through single-gene differential expression analysis.

### 4.10. Molecular Docking

The three-dimensional structures of the principal active ingredients were obtained from the PubChem database (https://pubchem.ncbi.nlm.nih.gov/, (accessed on 14 January 2026)) and saved in .sdf format. OpenBabelGUI 1.5.6 software facilitated the conversion of these structures to .pdb format. Additionally, the three-dimensional structures of key genes were retrieved from the PDB database (https://www.rcsb.org/, (accessed on 1 March 2026)) and also saved in .pdb format. Dehydration and hydrogenation treatments were applied to the primary active compounds and key genes using AutoDockTools 1.5.7, with all final files stored in .pdbqt format. Based on the structural features of the target protein, the region of the predicted active site is selected as the docking site, and the docking grid box is positioned within this region, with dimensions covering the entire protein. The primary active compounds and key genes were subsequently imported as ligands and receptors, respectively, for molecular docking using AutoDockTools version 1.5.7. The docking results were visualized with PYMOL 2.6.0 software.

### 4.11. RT-qPCR

Total RNA was extracted from mouse kidney tissue using Trizol lysis. The concentration and purity of RNA were assessed with a NanoDrop 2000 micro-volume nucleic acid analyzer (Thermo Fisher Scientific (China) Co., Ltd., Shanghai, China). RNA was reverse transcribed into cDNA according to the manufacturer’s instructions. Subsequently, quantitative PCR (qPCR) was conducted using a QuantStudio 5 real-time fluorescent quantitative PCR system (Thermo Fisher Scientific (China) Co., Ltd., Shanghai, China), with SYBR Green serving as the detection fluorophore. To standardize GAPDH gene expression, the 2^−ΔΔCt^ method was utilized to determine relative RNA expression. The primer sequences are provided in Table 2.

### 4.12. Western Blotting

Harvest an appropriate quantity of mouse kidney tissue into a grinding tube. Add a suitable amount of grinding beads along with 1 mL of pre-chilled RIPA cell lysis buffer, ensuring a final concentration of 1 mM PMSF and phosphatase inhibitors. Grind the tissue using a high-speed, low-temperature tissue grinder, and incubate on ice for 30 min to facilitate lysis. Following lysis, centrifuge the mixture at 4 °C at 12,000 rpm for 15 min. Carefully remove the supernatant, quantify the protein concentration using the BCA protein assay, aliquot the samples, and store them at −80 °C. Add 5× SDS loading buffer to the aliquots and denature the samples at 100 °C for 10 min. After loading, separate the proteins via SDS-PAGE. Transfer the target protein onto a PVDF membrane, block with 5% BSA for 2 h, wash three times with 1 × TBST, and incubate overnight at 4 °C with the primary antibody. The following day, remove the primary antibody, wash three times with 1 × TBST, incubate with the secondary antibody for 1.5 h, wash three times with 1 × TBST, scan the membrane using a multi-color fluorescence biomolecular imaging system (BIO-RAD, Hercules, CA, USA), and analyze the results with ImageJ 1.8.0 software.

### 4.13. Statistical Analysis

Statistical analyses were performed using GraphPad Prism 9.5.1. Each experiment was independently replicated at least three times, and data are presented as mean ± standard deviation (SD). For comparisons among multiple groups, one-way or two-way analysis of variance (ANOVA) was used, followed by Tukey’s multiple comparison test. *p* < 0.05 was considered statistically significant.

## 5. Conclusions

This study suggests that ZYBSW effectively alleviates CGN by improving renal function, attenuating pathological damage, and reducing inflammatory responses. Through integrated phytochemical analysis, network pharmacology, WGCNA, and experimental validation, we identified 419 chemical constituents in ZYBSW and provided evidence that its therapeutic effects are mediated via regulation of the MAPK signaling pathway through modulation of EGFR and DUSP1 expression. ZYBSW treatment was observed to inhibit EGFR expression, upregulate DUSP1, and reduce phosphorylation levels of ERK, JNK, and p38 in renal tissues. Molecular docking indicated that ZYBSW-derived compounds—Medicagenic acid, Gomisin, and Justicidin b—exhibited strong binding affinities to EGFR and DUSP1, suggesting their potential roles as active constituents. These findings highlight the multi-component and multi-target characteristics of ZYBSW in CGN treatment, offering a potential scientific basis for its clinical application in chronic kidney disease and providing insights into traditional Chinese medicine-based therapeutic strategies.

## 6. Patents

The manuscript contains a patent arising from patent application No. 202511978303.5.

## Figures and Tables

**Table 1:** The active compounds of ZYBSW.

Rank	Number	Ingredient Name	Formula	Betweenness
1	Z9	Gomisin	C_30_H_32_O_9_	1130.552
2	Z46	Medicagenic acid	C_30_H_46_O_6_	1030.9177
3	Z3	Gomisin_a	C_23_H_28_O_7_	956.95215
4	Z45	Bergamottin	C_21_H_22_O_4_	737.3513
5	Z44	Justicidin b	C_21_H_16_O_6_	687.66547

**Table 2:** Sequences of primer for qPCR.

Gene	Forward 5′ to 3′	Reverse 5′ to 3′
*TNF-α*	CCTCACACTCACAAACCACCAA	CTCCTGGTATGAGATAGCAAATCG
*IL-6*	CCCCAATTTCCAATGCTCTCC	CGCACTAGGTTTGCCGAGTA
*IL-1β*	GCTTCAGGCAGGCAGTATCA	AATGGGAACGTCACACACCA
*NR4A1*	GTGGCTTTGGTGATTGGATTG	TCAGTGATGAGGACCAGAGCG
*DUSP1*	ATCGTGCCCAACGCTGAACT	CGAAAACGCTTCATATCCTCCT
*STK16*	CCTCTTCCAGCATCCCAACAT	GGTCCTTCAGCCTTTCTATCTCATT
*CPT1B*	CTCTTCTGCCTTTACATCGTCTCC	AGAGACCCCGTAGCCATCATC
*CYP1A2*	GACACAGTCACCACAGCCATCA	TGGAAGCCATTCAGTGAGGTGT
*GAPDH*	CCTCGTCCCGTAGACAAAATG	TGAGGTCAATGAAGGGGTCGT

## Data Availability

The original contributions presented in this study are included in the article and Supplementary Material. Further inquiries can be directed to the corresponding authors.

## Supplementary Materials

The following supporting information can be downloaded at https://www.mdpi.com/article/10.3390/ph19050682/s1, Table S1. Component detection results.

## Abbreviations

ZYBSW	Zhuangyang Bushen Pill
CGN	Chronic glomerulonephritis
ADR	Adriamycin
CKD	Chronic kidney disease
ESRD	End-stage renal disease
TCM	Traditional Chinese medicine
LC-MS/MS	Liquid chromatography–tandem mass spectrometry
RT-qPCR	Quantitative reverse transcription polymerase chain reaction
H&E	Hematoxylin and eosin
mIHC	Multiplex immunohistochemistry
WGCNA	Weighted gene co-expression network analysis
SCr	Serum creatinine
BUN	Blood urea nitrogen
ALB	Albumin
ACR	Albumin-to-creatinine ratio
WBC	White blood cell count
RBC	Red blood cell
HCT	Hematocrit
HGB	Hemoglobin
PLT	Platelet count
DEGs	Differentially expressed genes
EGFR	Epidermal growth factor receptor
ERK	Extracellular regulated protein kinase
p-ERK	Phosphorylated extracellular regulated protein kinase
JNK	c-Jun N-terminal kinase
p-JNK	Phosphorylated c-Jun N-terminal kinase
p38	p38 mitogen-activated protein kinase
p-p38	Phosphorylated p38 mitogen-activated protein kinase
DUSP1	Dual-specificity phosphatase 1
MAPK	Mitogen-activated protein kinase
