# Supplementary material for: Zhuangyang Bushen Pill Attenuates Renal Injury in Chronic Glomerulonephritis by Suppressing the MAPK Signaling Pathway
Source: Pharmaceuticals (Basel). 2026 Apr 27;19(5):682. doi: 10.3390/ph19050682 (PMC13210072; doi:10.3390/ph19050682)
Supplement: Supplementary file 1 [file pharmaceuticals-19-00682-s001.zip › pharmaceuticals-4240728-supplementary.pdf]

Tabel S1 Component detection results

No.	Ingredient Name	m/z	Rt (min)	Formula	mode	CAS	Peak area
1	Bergapten	200.05	3.00	C12H8O4	[M-OH+H]+	484-20-8	4033533111
2	Pseudobaptigenin	281.25	11.15	C16H10O5	[M-H]-	90-29-9	2220645530
3	Petroselinic acid	563.50	10.74	C18H34O2	[2M-H]-	593-39-5	1462105549
4	Picrocrocin	314.17	3.91	C16H26O7	[M-OH+H]+	138-55-6	1330235209
5	Pinoresinol	342.14	0.77	C20H22O6	[M-OH+H]+	487-36-5	963751768.9
6	Gomisin_a	399.18	7.72	C23H28O7	[M-H2O+H]+	58546-54-6	959057795.7
7	Schizandrin	433.22	7.79	C24H32O7	[M+H]+	7432-28-2	810077931
8	Pyroglutamic acid	130.09	0.84	C5H7NO3	[M+H]+	4042-36-8	741565295.8
9	Hydroxyhydroquinone	127.04	1.10	C6H6O3	[M+H]+	533-73-3	724501196.2
10	Gomisin n	401.19	9.44	C23H28O6	[M+H]+	82467-52-5	717828056.6
11	Angeloylgomisin	501.25	8.08	C28H36O8	[M+H]+	66056-22-2	691706257.9
12	Icariin	677.25	5.62	C33H40O15	[M+H]+	489-32-7	626539944
13	Tutin	293.21	7.04	C15H18O6	[M-H]-	2571-22-4	530058255.2
14	3,4-dihydrocoumarin	166.09	1.64	C9H8O2	[M+NH4]+	119-84-6	490113622.8
15	Melilotoside	344.13	1.19	C15H18O8	[M+NH4]+	618-67-7	390154497.9
16	Tetrahydrocannabinol	315.25	8.02	C21H30O2	[M+H]+	1972-08-3	363990426.4

17	Senecionine	336.12	5.66	C18H25NO5	[M+H]+	130-01-8	345673280
18	Palmitaldehyde	301.24	10.60	C16H32O	[M+HCO3]-	629-80-1	326921231
19	1-cqa	353.09	1.27	C16H18O9	[M-H]-	1241-87-8	318763388.6
20	(s)-reticuline	330.17	4.40	C19H23NO4	[M+H]+	485-19-8	306640283.4
21	Biochanin a	285.07	5.84	C16H12O5	[M+H]+	491-80-5	288360324.6
22	Dihydrofolic acid	427.13	1.20	C19H21N7O6	[M-NH3+H]+	4033-27-6	287217628.9
23	Dalbergin	267.07	7.06	C16H12O4	[M-H]-	482-83-7	247810437.4
24	D-allothreonine	102.06	0.91	C4H9NO3	[M-H2O+H]+	24830-94-2	243171285.6
25	2-hydroxyadipic acid	145.05	1.16	C6H10O5	[M-H2O+H]+	18294-85-4	237451568.3
26	Asimilobine	250.09	0.89	C17H17NO2	[M-H2O+H]+	6871-21-2	235653764.5
27	Gomisin	554.24	8.22	C30H32O9	[M+NH4]+	58546-56-8	218660237
28	Demecolcine	328.19	4.29	C21H25NO5	[M-CO2+H]+	477-30-5	207759965.9
29	Pinostilbene	265.08	1.20	C15H14O3	[M+Na]+	42438-89-1	207716542.2
30	Madecassic acid	549.34	6.03	C30H48O6	[M+HCOO]-	18449-41-7	205059627.4
31	Chrysin	253.22	9.84	C15H10O4	[M-H]-	480-40-0	198303446.2
32	Synaptolepis factor k1	613.47	10.27	C36H54O8	[M-H]-	66268-94-8	189994860.6
33	Maleic acid	115.00	0.66	C4H4O4	[M-H]-	110-16-7	170608439
34	Luteone	353.07	0.65	C20H18O6	[M-H]-	41743-56-0	170274005.7

35	Schisantherin b	515.22	8.81	C28H34O9	[M+H]+	58546-55-7	169678165.4
36	Senegalensin	407.19	8.41	C25H28O5	[M-H]-	68236-11-3	164773448.4
37	Squalene	411.27	7.27	C30H50	[M+H]+	111-02-4	161060957.6
38	Dihydrotanshinone i	555.44	9.53	C18H14O3	[2M-H]-	87205-99-0	158801322.4
39	Licochalcone b	285.04	6.53	C16H14O5	[M-H]-	58749-23-8	158079179
40	Kaempferol	287.05	6.25	C15H10O6	[M+H]+	520-18-3	155967929.1
41	7-methylguanosine	297.24	9.17	C11H16N5O5+	[M-H]-	20244-86-4	147915436.9
42	Isoscopoletin	210.07	1.32	C10H8O4	[M+NH4]+	776-86-3	143260340.6
43	Demethylmedicarpin	255.07	5.48	C15H12O4	[M-H]-	61135-91-9	140337912.6
44	2'-deoxycytidine-5'-monophosphoric acid	325.09	4.24	C9H14N3O7P	[M+NH4]+	1032-65-1	133409031
45	Verbascoside	623.19	4.84	C29H36O15	[M-H]-	61276-17-3	133122735.3
46	Astragalin	447.09	4.87	C21H20O11	[M-H]-	480-10-4	132751816.4
47	Uric acid	152.04	0.65	C5H4N4O3	[M-OH+H]+	69-93-2	132226493.4
48	eicosa-11,14-dienoic acid	307.26	10.93	C20H36O2	[M-H]-	2091-39-6	130715249.9
49	Quercetin	303.05	5.81	C15H10O7	[M+H]+	117-39-5	129872535.8
50	Picrotin	309.21	7.10	C15H18O7	[M-H]-	21416-53-5	118104286.9
51	Eriodictyol chalcone	269.05	7.48	C15H12O6	[M-H2O-H]-	14917-41-0	114244094.2
52	Trigonelline	138.05	0.93	C7H7NO2	[M+H]+	535-83-1	113295313.6

53	Scopolin	353.09	1.64	C16H18O9	[M-H]-	531-44-2	112965726.3
54	Terephthalic acid	149.02	9.09	C8H6O4	[M-H2O+H]+	100-21-0	112568854.9
55	Medicocarpin	431.14	0.78	C22H24O9	[M-H]-	52766-70-8	107303925.1
56	2-furoic acid	111.01	0.63	C5H4O3	[M-H]-	88-14-2	107011307.6
57	Agaric_acid	415.27	8.11	C22H40O7	[M-H]-	666-99-9	105862984.9
58	7,4'-dihydroxyflavone	253.05	7.79	C15H10O4	[M-H]-	2196-14-7	102499764.2
59	Strictosamide	455.22	7.47	C26H30N2O8	[M-CO2+H]+	23141-25-5	99691391.97
60	Cyanidin 3-o-sophoroside	612.16	10.88	[C27H31O16]+	[M+H]+	38820-68-7	98842802.68
61	Isoetin	303.05	4.73	C15H10O7	[M+H]+	1621-84-7	98437686.32
62	Solavetivone	219.17	9.61	C15H22O	[M+H]+	54878-25-0	96817718.32
63	Angeloylgomisin q	548.25	7.56	C29H38O9	[M+NH4]+	72561-28-5	96698190.67
64	Quercetin 3-sambubioside	597.15	4.54	C26H28O16	[M+H]+	83048-35-5	96658546.66
65	Eupatilin	343.21	4.31	C18H16O7	[M-H]-	22368-21-4	94285670.16
66	4-hydroxybenzoic acid	137.02	2.07	C7H6O3	[M-H]-	99-96-7	91332404.69
67	Stachydrine	144.10	0.83	C7H13NO2	[M+H]+	471-87-4	89174592.5
68	3-o-p-coumaroylquinic acid	337.09	1.63	C16H18O8	[M-H]-	32451-86-8	88422160.39
69	1-methyladenosine	262.09	1.04	C11H15N5O4	[M-H2O-H]-	15763-06-1	84093996.42
70	Aflatoxin b1	313.07	7.35	C17H12O6	[M+H]+	1162-65-8	82966232.41

71	Gardenin	341.14	4.25	C19H18O7	[M-H2O+H]+	2798-20-1	81373192.22
72	3-indoleacetonitrile	139.99	7.47	C10H8N2	[M-OH+H]+	771-51-7	79193426.41
73	Cinchonine	293.21	9.36	C19H22N2O	[M-H]-	118-10-5	78408760.12
74	Isorhamnetin	315.05	6.30	C16H12O7	[M-H]-	480-19-3	77535786.36
75	Daphnin	323.07	4.26	C15H16O9	[M-H2O+H]+	486-55-5	77308346.31
76	(s)-3',4',5,7-tetrahydroxy-5',8-diprenylflavanon e	425.21	9.50	C25H28O6	[M+H]+	124596-89-0	76844739.12
77	Tyramine	120.08	1.64	C8H11NO	[M-H2O+H]+	51-67-2	76124236.66
78	Rutin	611.17	4.61	C27H30O16	[M+H]+	153-18-4	74415176.69
79	Echinacoside	804.30	4.41	C35H46O20	[M+NH4]+	82854-37-3	73920114.12
80	Alliin	195.08	2.99	C6H11NO3S	[M+NH4]+	556-27-4	72523574.46
81	Osajin	405.35	5.15	C25H24O5	[M+H]+	482-53-1	72214602.72
82	Astaxanthin	614.49	9.99	C40H52O4	[M+NH4]+	472-61-7	71481445.47
83	Bursehernin	388.17	4.66	C21H22O6	[M+NH4]+	40456-51-7	70918571.98
84	Ginsenoside re	991.54	5.15	C48H82O18	[M+HCOO]-	52286-59-6	67352021.14
85	Isoflavone	205.07	0.94	C15H10O2	[M-H2O+H]+	574-12-9	66122907.69
86	Pheophorbide a	593.28	10.52	C35H36N4O5	[M+H]+	15664-29-6	63371294.97
87	Pratensein	299.26	9.60	C16H12O6	[M-H]-	2284-31-3	63186243.54
88	Pyrethrin i	373.20	9.57	C21H28O3	[M+HCOO]-	121-21-1	60423043.66

89	Coumarin	130.02	0.65	C9H6O2	[M-NH3+H]+	91-64-5	60202580.29
90	Wighteone	295.15	0.84	C20H18O5	[M-CO2+H]+	51225-30-0	60173347.16
91	4-guanidinobutanoic acid	146.09	0.84	C5H11N3O2	[M+H]+	463-00-3	59360210.71
92	2-hydroxybenzyl alcohol	107.05	4.49	C7H8O2	[M-H2O+H]+	90-01-7	59163839.24
93	9-Methoxycamptothecine	377.08	1.00	C21H18N2O5	[M-H]-	39026-92-1	59088515.45
94	Velloquercetin	367.11	1.09	C20H16O7	[M-H]-	139955-63-8	59032661.72
95	(+)-calycanthine	347.10	0.83	C22H26N4	[M+H]+	595-05-1	58182223.41
96	Cephaeline	450.26	7.78	C28H38N2O4	[M-NH3+H]+	483-17-0	57941702.58
97	Trans-2-butene-1,4-dicarboxylic acid	145.05	5.16	C6H8O4	[M+H]+	4436-74-2	57559242.34
98	Peonidin 3-rutinoside	610.21	11.38	C28H33O15	[M+H]+	27539-32-8	56450310.43
99	Eucalyptin	307.19	6.49	C19H18O5	[M-H2O-H]-	3122-88-1	56251392.84
100	Glycitin	447.13	4.69	C22H22O10	[M+H]+	40246-10-4	55929176.28
101	Asarone	209.15	4.98	C12H16O3	[M+H]+	2883-98-9	55224122.03
102	Oleuroside	539.14	0.75	C25H32O13	[M-H]-	116383-31-4	54824788.34
103	3-o-feruloylquinic acid	367.10	2.18	C17H20O9	[M-H]-	1899-29-2	54576426.88
104	2-tridecanone	243.20	6.13	C13H26O	[M+HCOO]-	593-08-8	53628857.84
105	N-trans-feruloyltyramine	314.13	5.64	C18H19NO4	[M+H]+	66648-43-9	52343645.59
106	Chlorogenic acid	353.31	10.05	C16H18O9	[M-H]-	327-97-9	50926327.63

107	Hypericin	503.33	6.32	C30H16O8	[M-H]-	548-04-9	50860722.46
108	Hydrocinnamic acid	151.07	3.83	C9H10O2	[M+H]+	501-52-0	50502660.84
109	Indole-3-carboxaldehyde	146.06	3.21	C9H7NO	[M+H]+	487-89-8	49947012.21
110	Licodione	271.23	7.29	C15H12O5	[M-H]-	61153-76-2	48949200.05
111	Hymatoxin a	414.13	3.59	C20H29O7S-	[M+H]+	109621-33-2	48898836.39
112	Rutarin	447.12	4.70	C20H24O10	[M+Na]+	20320-81-4	48820876.14
113	Ellagic acid	301.03	6.33	C14H6O8	[M-H]-	476-66-4	48648589.11
114	Oripavine	280.13	0.81	C18H19NO3	[M-H2O+H]+	467-04-9	47354865.77
115	Gypsogenin	471.34	7.66	C30H46O4	[M+H]+	639-14-5	47123008.41
116	Harmine	195.09	6.88	C13H12N2O	[M-H2O+H]+	442-51-3	45988140.56
117	Alpinetin	269.08	6.89	C16H14O4	[M-H]-	36052-37-6	44796472.72
118	Isoformononetin	269.08	7.47	C16H12O4	[M+H]+	486-63-5	44201893.36
119	Aesculetin	179.11	7.54	C9H6O4	[M+H]+	305-01-1	44193584.91
120	Podophyllotoxin, glucoside	559.19	8.44	C28H32O13	[M-H2O+H]+	16481-54-2	44140254.57
121	Sumiki's acid	143.04	4.23	C6H6O4	[M+H]+	6338-41-6	43523407.8
122	Isorhamnetin 3-galactoside	479.11	5.00	C22H22O12	[M+H]+	6743-92-6	43390083.96
123	Sesaminol	409.20	8.62	C20H18O7	[M+K]+	74061-79-3	42135715.46
124	Beta-mangostin	425.22	9.50	C25H28O6	[M+H]+	20931-37-7	41881654.3

125	Cianidanol	291.09	3.83	C15H14O6	[M+H]+	154-23-4	41870934.36
126	Betulinic acid	501.35	10.17	C30H48O3	[M+HCOO]-	472-15-1	41819191.92
127	Vicine	288.11	1.19	C10H16N4O7	[M-OH+H]+	152-93-2	41626044.76
128	Rhoifolin	579.18	1.74	C27H30O14	[M+H]+	17306-46-6	41237803.97
129	Alpha-allocryptopine	430.15	0.78	C21H23NO5	[M+HCO3]-	485-91-6	40226396.81
130	2-oxoadipic acid	143.03	1.26	C6H8O5	[M-H2O+H]+	3184-35-8	40121177.72
131	Swertiamarin	392.15	1.68	C16H22O10	[M+NH4]+	17388-39-5	40076622.5
132	Cinnamic acid	166.09	1.35	C9H8O2	[M+NH4]+	140-10-3	39793107.47
133	5-hydroxyindoleacetate	192.14	1.89	C10H9NO3	[M+H]+	54-16-0	39243680.32
134	Kaempferol 3-sophoroside 7-rhamnoside	713.23	10.77	C33H40O20	[M-CO2+H]+	93098-79-4	38576409.57
135	Diethylphosphate	155.04	2.99	C4H11O4P	[M+H]+	598-02-7	38402766.69
136	Salidroside	345.12	2.88	C14H20O7	[M+HCOO]-	10338-51-9	38336804.55
137	Karanjin	293.21	8.06	C18H12O4	[M+H]+	521-88-0	38038828.92
138	Patuletin	393.04	0.70	C16H12O8	[M+HCO3]-	519-96-0	37680292.19
139	Ginsenoside	845.50	5.93	C42H72O14	[M+HCOO]-	52286-58-5	37284197.5
140	Garbanzol	271.06	6.05	C15H12O5	[M-H]-	1226-22-8	37224663.78
141	Isocorypalmine	386.16	2.87	C20H23NO4	[M+HCOO]-	483-34-1	36838598.44
142	Alpha-bixin	455.21	10.42	C25H30O4	[M+HCO3]-	6983-79-5	36665916.67

143	Coptisine	304.10	0.76	C19H14NO4+	[M-OH+H]+	3486-66-6	36470578.94
144	Hymecromone	177.05	4.27	C10H8O3	[M+H]+	90-33-5	35566569.3
145	Vinorine	318.15	2.90	C21H22N2O2	[M-NH3+H]+	34020-07-0	35393971.1
146	Arctiin	579.21	4.96	C27H34O11	[M+HCOO]-	20362-31-6	35105393.48
147	Ceanothic acid	485.27	8.54	C30H46O5	[M-H]-	21302-79-4	34096164.61
148	Patulitrin	533.21	5.89	C22H22O13	[M+K]+	19833-25-1	33712372.61
149	Licoisoflavone a	372.12	1.18	C20H18O6	[M+NH4]+	66056-19-7	33646682.81
150	6alpha-hydroxymaackiain	301.11	6.82	C16H12O6	[M+H]+	14602-93-8	33460963.76
151	Biochanin a 7-(6-malonylglucoside)	533.13	5.03	C25H24O13	[M+H]+	34232-17-2	32814023.96
152	(+)-epicatechin	289.07	3.85	C15H14O6	[M-H]-	35323-91-2	32119433.65
153	Quercetagetin-7-O-glucoside	519.19	5.41	C21H20O13	[M+K]+	548-75-4	32053145.78
154	Salvigenin	285.11	7.62	C18H16O6	[M-CO2+H]+	19103-54-9	31547752.93
155	Diphyllin	381.10	0.74	C21H16O7	[M+H]+	22055-22-7	30001051.91
156	Atractylenolide iii	231.14	8.13	C15H20O3	[M-H2O+H]+	73030-71-4	29833096.43
157	Caffeic acid	163.04	4.85	C9H8O4	[M-H2O+H]+	331-39-5	29691546.5
158	Luteolin	287.06	4.97	C15H10O6	[M+H]+	491-70-3	29590994.13
159	Combretol	427.21	3.60	C20H20O8	[M+K]+	5084-19-5	29503886.2
160	Pinocembrin	255.07	7.12	C15H12O4	[M-H]-	480-39-7	29502888.56

161	Methyl 2-hydroxybenzoate	197.05	4.80	C8H8O3	[M+HCOO]-	119-36-8	29410703.09
162	8-prenylnaringenin	341.14	5.76	C20H20O5	[M+H]+	53846-50-7	29241151.82
163	Velutin	313.22	6.43	C17H14O6	[M-H]-	25739-41-7	28985390.76
164	Quinic acid	383.11	0.71	C7H12O6	[2M-H]-	77-95-2	28938719.94
165	Daphnetol	177.02	5.12	C9H6O4	[M-H]-	486-35-1	28314172.13
166	Isotirumalin	401.16	4.95	C22H24O7	[M+H]+	119736-72-0	28267863.31
167	Secologanin	433.14	4.20	C17H24O10	[M+HCOO]-	19351-63-4	28124180.21
168	4-aminophenol	110.06	0.84	C6H7NO	[M+H]+	123-30-8	27546312
169	Isovitexin	431.10	5.17	C21H20O10	[M-H]-	38953-85-4	27543476.97
170	Sinapic acid	207.14	4.81	C11H12O5	[M-H2O+H]+	530-59-6	27211578.1
171	Eupatolide	293.18	10.66	C15H20O3	[M+HCOO]-	6750-25-0	27049582.63
172	Cis-melilotoside	325.09	4.21	C15H18O8	[M-H]-	2446-60-8	26817082.09
173	Betulin	443.39	6.26	C30H50O2	[M+H]+	473-98-3	26396936.22
174	Vanillic acid	167.04	4.87	C8H8O4	[M-H]-	121-34-6	26330424.47
175	Glyceocarpin	323.13	4.95	C20H20O5	[M-H2O+H]+	77979-22-7	25927680.84
176	Acacetin 7-[apiosyl(1->6)-glucoside]	577.16	5.06	C27H30O14	[M-H]-	239106-94-6	25417295.39
177	Taurodeoxycholate	480.28	9.48	C26H45NO6S	[M-H2O-H]-	516-50-7	25138341.06
178	Chebulic_acid	355.10	4.39	C14H12O11	[M-H]-	23725-05-5	25088046.48

179	3-hydroxyflavone	237.06	7.31	C15H10O3	[M-H]-	577-85-5	25010405.72
180	Ginsenoside rd	991.54	6.24	C48H82O18	[M+HCOO]-	52705-93-8	24942850.24
181	Norepinephrine	187.11	0.82	C8H11NO3	[M+NH4]+	51-41-2	24722307.54
182	Dihydrodaidzein	255.12	4.86	C15H12O4	[M-H]-	17238-05-0	24163407.19
183	Hemigossypol	521.21	9.50	C15H16O4	[2M+H]+	40817-07-0	24161310.7
184	Beta-elemolic acid	439.35	10.00	C30H48O3	[M-H2O+H]+	28282-27-1	24029056.03
185	Lignans	827.26	4.57	C22H22O8	[2M-H]-	477-47-4	23964629.01
186	Pollenitin	317.06	6.34	C16H12O7	[M+H]+	24487-56-7	23766319.24
187	Resveratrol	227.20	9.62	C14H12O3	[M-H]-	501-36-0	23588416.43
188	Pseudoginsenoside rt5	635.53	10.22	C36H62O10	[M-H2O-H]-	98474-78-3	23545088.08
189	Vasicine	189.10	2.05	C11H12N2O	[M+H]+	6159-55-3	23515012.72
190	Coreopsin	867.25	5.49	C21H22O10	[2M-H]-	499-29-6	23507376.5
191	5,6,2-trimethoxyflavone	313.11	7.07	C18H16O5	[M+H]+	16266-97-0	23491596.68
192	Serotonin	160.08	4.07	C10H12N2O	[M-NH3+H]+	50-67-9	23367688.33
193	Davallioside a	519.18	5.41	C25H29NO12	[M-OH+H]+	131747-15-4	23294514.64
194	1-dehydroxy-23-deoxojessic acid	469.34	9.65	C31H50O3	[M-H]-	149252-87-9	23243246.31
195	Vitexin	433.12	5.27	C21H20O10	[M+H]+	3681-93-4	23131747.43
196	Lycorine	305.15	10.22	C16H17NO4	[M+NH4]+	476-28-8	22764353.39

197	Nepetin	315.16	5.62	C16H12O7	[M-H]-	520-11-6	22745934.82
198	Kaempferol 7-o-glucoside	449.11	3.82	C21H20O11	[M+H]+	16290-07-6	22653403.92
199	Castanin	282.08	1.01	C17H14O5	[M-OH+H]+	550-79-8	22404283.16
200	2-methylguanosine	281.14	0.97	C11H15N5O5	[M-OH+H]+	2140-77-4	22294832.56
201	Chrysoplenol d	405.09	6.71	C18H16O8	[M+HCOO]-	14965-20-9	22185163.48
202	Gomisin s	401.19	6.79	C23H30O7	[M-H2O+H]+	119239-49-5	22052573.03
203	Leucocyanidin	287.06	5.18	C15H14O7	[M-H2O-H]-	480-17-1	22046207.52
204	Sakuranetin	287.09	5.49	C16H14O5	[M+H]+	2957-21-3	21965698.8
205	Wistin	459.13	0.72	C23H24O10	[M-H]-	19046-26-5	21965006.57
206	O-feruloylquinate	367.10	3.41	C17H20O9	[M-H]-	2613-86-7	21887256.65
207	Geranyl acetate	393.30	7.58	C12H20O2	[2M+H]+	105-87-3	21869430.17
208	Rotenone	787.28	4.37	C23H22O6	[2M-H]-	83-79-4	21524407.67
209	3,5-dimethoxyphenol	155.07	5.09	C8H10O3	[M+H]+	500-99-2	21403340.99
210	Violaxanthin	584.43	9.89	C40H56O4	[M-OH+H]+	126-29-4	20866153.52
211	Calycosin	283.06	6.46	C16H12O5	[M-H]-	20575-57-9	20842241.95
212	Amygdaloside	456.16	0.80	C20H27NO11	[M-H]-	29883-15-6	20797562.83
213	Cotinine	221.07	1.16	C10H12N2O	[M+HCOO]-	486-56-6	20767730.83
214	Tiliroside	595.14	5.58	C30H26O13	[M+H]+	20316-62-5	20614087.38

215	Vinblastine	767.44	7.40	C46H58N4O9	[M-CO2+H]+	865-21-4	20449418
216	(+)-camphor	153.13	4.72	C10H16O	[M+H]+	464-49-3	20435499.73
217	Betunal	424.37	9.63	C30H48O2	[M-OH+H]+	13159-28-9	20327867.46
218	Polygalic	487.15	4.04	C29H44O6	[M-H]-	1260-04-4	20063348.09
219	2-pyrocatechuic acid	153.02	3.75	C7H6O4	[M-H]-	303-38-8	20055601.55
220	Spinacetin	364.10	0.73	C17H14O8	[M+NH4]+	3153-83-1	20044648.57
221	Auriculoside	431.22	7.06	C22H26O10	[M-H2O-H]-	75871-96-4	19870440.56
222	Aurantiamide	403.20	6.95	C25H26N2O3	[M+H]+	58115-31-4	19825757.73
223	Tricin	329.23	4.97	C17H14O7	[M-H]-	520-32-1	19700177.43
224	5-hydroxyconiferaldehyde	239.06	9.77	C10H10O4	[M+HCOO]-	249647-14-1	19599807.04
225	5-hydroxyferulic acid	211.06	1.21	C10H10O5	[M+H]+	1782-55-4	19518747.39
226	Ergokryptine	620.31	6.07	C32H41N5O5	[M+HCOO]-	511-09-1	19287691.48
227	Gf vi	767.50	5.81	C42H72O13	[M-H2O+H]+	52286-74-5	19197335.87
228	Disinapoyl sucrose	772.27	5.02	C34H42O19	[M+NH4]+	139891-98-8	19155971.68
229	Pisiferic acid	317.22	4.00	C20H28O3	[M+H]+	67494-15-9	19026497.36
230	Artelastochromene	531.33	7.68	C30H30O6	[M+HCOO]-	182052-09-1	18891237
231	Nobiletin	403.14	7.22	C21H22O8	[M+H]+	478-01-3	18732803.63
232	Docosanedioic acid	369.30	10.03	C22H42O4	[M-H]-	505-56-6	18704756.46

233	Rishitin	223.17	7.55	C14H22O2	[M+H]+	18178-54-6	18646602.69
234	Myricetin 3-galactoside	481.10	4.48	C21H20O13	[M+H]+	15648-86-9	18579433.33
235	3,4-methylenedioxybenzoic acid	167.07	8.02	C8H6O4	[M+H]+	94-53-1	18396273.4
236	Dehydroferreirin	301.07	6.36	C16H12O6	[M+H]+	32884-35-8	18367866.67
237	(-)-maackiain	285.07	6.87	C16H12O5	[M+H]+	2035-15-6	18229274.76
238	Neoliquiritin 2'-apioside	533.14	5.03	C26H30O13	[M-H2O+H]+	135432-48-3	18096705.36
239	Uralenol	353.10	7.04	C20H18O7	[M-H2O+H]+	139163-15-8	17963551.27
240	Lecanoricacid	317.06	5.23	C16H14O7	[M-H]-	480-56-8	17840913.27
241	Morelloflavone	555.04	0.88	C30H20O11	[M-H]-	16851-21-1	17723208.48
242	Isoquercitrin	465.10	4.53	C21H20O12	[M+H]+	482-35-9	17467473.04
243	Xanthosine	265.06	0.66	C10H12N4O6	[M-H2O-H]-	146-80-5	17395269.32
244	4-hydroxycinnamoylagmatine	277.16	3.94	C14H20N4O2	[M+H]+	7295-86-5	17390582.11
245	2-phenylpropionate	151.07	2.04	C9H10O2	[M+H]+	492-37-5	17082924.53
246	Soyasapogenol b	459.38	5.15	C30H50O3	[M+H]+	595-15-3	17053153.16
247	Propapyriogenin a2	483.27	7.41	C30H44O5	[M-H]-	72933-74-5	16853403.97
248	Aristolochic acid c	328.15	1.57	C16H9NO7	[M+H]+	4849-90-5	16682456.91
249	Laserpitin	434.21	4.54	C25H38O7	[M-NH3+H]+	7067-12-1	16583697.97
250	4-hydroxycinnamic acid	147.04	4.33	C9H8O3	[M-H2O+H]+	501-98-4	16493659.81

251	Orcinol	142.09	1.81	C7H8O2	[M+NH4]+	504-15-4	16410324.76
252	Norgalantamine	274.14	0.83	C16H19NO3	[M+H]+	41303-74-6	16345749.21
253	Gibberellin a6	328.22	5.29	C19H22O6	[M-NH3-H]-	19147-78-5	16267173.12
254	Protopine	371.16	8.02	C20H19NO5	[M+NH4]+	130-86-9	15876179.3
255	Stepharine	298.14	4.20	C18H19NO3	[M+H]+	2810-21-1	15854104.83
256	Coniferyl alcohol	163.08	4.87	C10H12O3	[M-H2O+H]+	32811-40-8	15832669.06
257	1-phenyl-1,2-propanedione	149.06	1.66	C9H8O2	[M+H]+	579-07-7	15736242.03
258	Pluviatolide	711.24	0.78	C20H20O6	[2M-H]-	28115-68-6	15736143.1
259	Kaempferide	299.06	6.29	C16H12O6	[M-H]-	491-54-3	15724621.6
260	Boldine	328.15	4.50	C19H21NO4	[M+H]+	476-70-0	15609267.91
261	(-)-arctigenin	371.12	1.28	C21H24O6	[M-H]-	7770-78-7	15525728.9
262	Glycyrrhetinic acid	453.34	7.31	C30H46O4	[M-H2O+H]+	1449-05-4	15371425.14
263	Ayanin	301.11	5.59	C18H16O7	[M-CO2+H]+	572-32-7	15206764.15
264	Sanguinarine	315.09	5.93	[C20H14NO4]+	[M-H2O+H]+	2447-54-3	15173322.81
265	3,4-dihydroxyhydrocinnamic acid	163.04	5.51	C9H10O4	[M-H2O-H]-	1078-61-1	15112457.61
266	Tyrosol	156.10	3.61	C8H10O2	[M+NH4]+	501-94-0	15106518.38
267	Harmol	397.17	9.30	C12H10N2O	[2M+H]+	487-03-6	15037207.44
268	Aromadendrin	287.06	5.60	C15H12O6	[M-H]-	480-20-6	14936451.24

269	Ginsenoside f3	815.47	9.92	C41H70O13	[M+HCOO]-	62025-50-7	14905711.07
270	Oleanolic acid	911.72	9.98	C30H48O3	[2M-H]-	508-02-1	14626161.64
271	Niacinamide	123.05	5.80	C6H6N2O	[M+H]+	98-92-0	14478042.02
272	Ginsenoside ro	955.48	5.31	C48H76O19	[M-H]-	34367-04-9	14449150.62
273	Quercetin 3-glucosyl-(1->2)-galactoside	627.16	3.76	C27H30O17	[M+H]+	18609-17-1	14306718.84
274	2,3,4-trihydroxybenzoic acid	169.01	1.34	C7H6O5	[M-H]-	610-02-6	14102432.02
275	Justicidin b	727.20	0.84	C21H16O6	[2M-H]-	17951-19-8	13900044.47
276	Malvidin 3-glucoside-4-vinylphenol	593.16	11.38	C31H29O13	[M-NH3+H]+	388089-39-2	13878837.36
277	Propranolol	277.15	0.84	C16H21NO2	[M+NH4]+	525-66-6	13818952.47
278	Glycitein	283.06	4.32	C16H12O5	[M-H]-	40957-83-3	13601099.37
279	Hederagenin	471.34	7.89	C30H48O4	[M-H]-	465-99-6	13561295.3
280	Ononin	429.12	1.17	C22H22O9	[M-H]-	486-62-4	13486425.67
281	Taxifolin	303.05	4.76	C15H12O7	[M-H]-	480-18-2	13480918.55
282	Uralsaponin d	849.53	9.65	C42H58O18	[M-H]-	1262489-44-0	13445987.09
283	Calenduloside e	693.39	5.82	C36H56O9	[M+HCO3]-	26020-14-4	13389928.3
284	2'-hydroxygenistein	267.03	4.97	C15H10O6	[M-H2O-H]-	1156-78-1	13383436.97
285	6-hydroxydaidzein	271.06	6.46	C15H10O5	[M+H]+	17817-31-1	13299537.35
286	Dihydrotricetin	285.04	7.23	C15H12O7	[M-H2O-H]-	81398-31-4	13195402.15

287	N-(p-hydroxyphenyl)ethyl p-hydroxycinnamide	282.11	5.54	C17H17NO3	[M-H]-	36417-86-4	13171710.06
288	Galantamine	310.14	4.18	C17H21NO3	[M+Na]+	357-70-0	13060644.31
289	(e)-methyl ester 3-phenyl-2-propenoic acid	163.08	4.65	C10H10O2	[M+H]+	103-26-4	12852444.49
290	Pechueloic acid	249.11	4.54	C15H20O3	[M+H]+	83161-56-2	12846024.5
291	Regaloside c	461.16	4.54	C18H24O11	[M+HCOO]-	117591-85-2	12729562.05
292	Apigenin	271.06	6.18	C15H10O5	[M+H]+	520-36-5	12669953.11
293	Scopoletin	193.05	5.02	C10H8O4	[M+H]+	92-61-5	12559800.64
294	Indoleacetate	174.06	4.96	C10H9NO2	[M-H]-	87-51-4	12405983.55
295	Decuroside iii	554.17	10.46	C26H34O14	[M-NH3+H]+	96638-81-2	12220637.64
296	Eleganin	435.16	5.36	C22H26O9	[M+H]+	57498-84-7	12177262.48
297	Kaempferol 3-rungioside	593.15	4.90	C27H30O15	[M-H]-	28447-29-2	12022841.99
298	Lupanine	249.11	5.05	C15H24N2O	[M+H]+	550-90-3	11945675.44
299	Tryptamine	183.09	4.51	C10H12N2	[M+Na]+	61-54-1	11850049.72
300	Rhamnetin	315.06	6.56	C16H12O7	[M-H]-	90-19-7	11834405.8
301	Brassylic acid	243.16	4.61	C13H24O4	[M-H]-	505-52-2	11792646.65
302	Munduserone	365.10	6.11	C19H18O6	[M+Na]+	3564-85-0	11745541.7
303	Homoeriodictyol chalcone	303.08	4.69	C16H14O6	[M+H]+	25515-47-3	11682248.55
304	Angoletin	299.22	6.06	C18H20O4	[M-H]-	76444-55-8	11672468.26

305	Silibinin	481.12	5.96	C25H22O10	[M-H]-	22888-70-6	11533573.54
306	Anabasine	185.09	9.21	C10H14N2	[M+Na]+	494-52-0	11228834.83
307	Epifriedelanol	427.23	5.16	C30H52O	[M-H]-	16844-71-6	11165797.86
308	Biotin	245.18	4.11	C10H16N2O3S	[M+H]+	58-85-5	11019285.69
309	Ginsenoside rg3	785.51	5.81	C42H72O13	[M+H]+	14197-60-5	11012169.17
310	Sesamolin	393.10	5.86	C20H18O7	[M+Na]+	526-07-8	11011578.22
311	Sinapyl alcohol	193.09	3.79	C11H14O4	[M-H2O+H]+	537-33-7	10955327.64
312	Hydrastine	384.11	1.60	C21H21NO6	[M+H]+	118-08-1	10855764.44
313	Cirsimaritin	271.10	6.88	C17H14O6	[M-CO2+H]+	6601-62-3	10755888.09
314	Syringin	390.17	3.80	C17H24O9	[M+NH4]+	118-34-3	10741562.49
315	Sulcatone	171.10	4.19	C8H14O	[M+HCOO]-	110-93-0	10736904.48
316	Hydroxyacetone	147.07	1.17	C3H6O2	[2M-H]-	116-09-6	10620412.63
317	Traxillaside	625.22	4.69	C28H36O12	[M+HCO3]-	149415-62-3	10595590.06
318	Canadine	340.15	4.64	C20H21NO4	[M+H]+	5096-57-1	10581438.08
319	Paeoniflorin	525.17	4.53	C23H28O11	[M+HCOO]-	23180-57-6	10580186.12
320	Capsorubin	557.43	8.55	C40H56O4	[M-CO2+H]+	470-38-2	10569252.66
321	Quercetin 3-o-glucuronide	479.09	4.78	C21H18O13	[M+H]+	22688-79-5	10349255.38
322	4-acetamidobutanoate	144.05	3.29	C6H11NO3	[M-H]-	3025-96-5	10211255.08

323	Ferulate	193.05	5.80	C10H10O4	[M-H]-	537-98-4	10176894.13
324	Datiscin	593.16	4.90	C27H30O15	[M-H]-	16310-92-2	10119520.14
325	Angoroside	783.45	10.37	C36H48O19	[M-H]-	115909-22-3	10105529.11
326	8-hydroxypinoresinol 8-glucoside	535.19	7.82	C26H32O12	[M-H]-	81495-71-8	10090285.89
327	Acetophenone	165.06	3.80	C8H8O	[M+HCOO]-	98-86-2	9977377.156
328	Beta-ionone	253.14	5.14	C13H20O	[M+HCO3]-	79-77-6	9886843.575
329	Ginsenoside rk3	603.43	5.17	C36H60O8	[M-H2O+H]+	364779-15-7	9882589.513
330	Pinoquercetin	317.06	5.00	C16H12O7	[M+H]+	491-49-6	9870352.816
331	5-hydroxypseudobaptigenin	343.25	8.20	C16H10O6	[M+HCOO]-	40624-03-1	9793105.139
332	Crassicauline a	644.34	6.37	C35H49NO10	[M+H]+	79592-91-9	9782728.682
333	Bancroftinone	228.19	7.85	C11H14O4	[M+NH4]+	14964-98-8	9776190.746
334	Palmatine	397.15	7.63	[C21H22NO4]+	[M+HCOO]-	3486-67-7	9698343.193
335	Cryptotanshinone	319.13	8.76	C19H20O3	[M+Na]+	35825-57-1	9679856.705
336	Leucopelargonidin	291.09	2.43	C15H14O6	[M+H]+	98919-66-5	9373735.984
337	Dicrocin	633.26	6.75	C32H44O14	[M-H2O-H]-	57710-64-2	9315561.754
338	Matairesinol	340.10	1.19	C20H22O6	[M-NH3-H]-	580-72-3	9313461.063
339	Esculentic acid (diplazium)	533.35	7.04	C30H48O5	[M+HCOO]-	464-92-6	9280572.768
340	Ginsenoside rh2	667.45	10.28	C36H62O8	[M+HCOO]-	78214-33-2	9163588.019

341	Peonidin 3-rhamnoside 5-glucoside	610.21	10.34	C28H33O15+	[M+H]+	53859-11-3	9159454.078
342	Panasenoside	628.18	11.28	C27H30O16	[M+NH4]+	31512-06-8	9073133.319
343	Umbelliferone	180.10	3.76	C9H6O3	[M+NH4]+	93-35-6	9061040.582
344	Prunetin	283.06	7.24	C16H12O5	[M-H]-	552-59-0	9001702.717
345	Pinusolide	693.26	6.00	C21H30O4	[2M+H]+	31685-80-0	8963471.586
346	Phaseollin	279.14	2.14	C20H18O4	[M-CO2+H]+	13401-40-6	8373564.517
347	Formononetin	267.07	4.82	C16H12O4	[M-H]-	485-72-3	8190809.303
348	Delphinidin 3-rutinoside	595.15	11.40	[C27H31O16]+	[M-NH3+H]+	15674-58-5	8073458.684
349	Pomiferin	419.15	8.17	C25H24O6	[M-H]-	572-03-2	8065831.788
350	Leucine-betaxanthin	683.21	6.76	C34H34N2O16	[M-CO2+H]+	32074-65-0	7641952.953
351	Isorhapontin	465.14	4.97	C21H24O9	[M+HCOO]-	32727-29-0	7555850.265
352	Gibberellin a51	377.16	4.93	C19H24O5	[M+HCOO]-	56978-14-4	7504338.978
353	Kaempferol-3-rutinoside	595.16	4.81	C27H30O15	[M+H]+	17650-84-9	7467678.758
354	Entadamide a beta-d-glucopyranoside	307.11	1.45	C12H21NO7S	[M-OH+H]+	138916-58-2	7419832.561
355	Soyasaponin i	899.54	5.91	C48H78O18	[M-CO2+H]+	51330-27-9	7415662.135
356	Olivetol	225.11	1.42	C11H16O2	[M+HCOO]-	500-66-3	7273346.954
357	Morin	301.07	6.12	C15H10O7	[M-H]-	480-16-0	7233558.801
358	Thymol	149.02	4.60	C10H14O	[M-H]-	89-83-8	7149235.02

359	Betanidin	387.08	0.77	C18H16N2O8	[M-H]-	2181-76-2	7121352.953
360	Poricoic acid b	485.33	8.36	C30H44O5	[M+H]+	137551-39-4	7106512.566
361	Syringic acid	179.04	5.52	C9H10O5	[M-H2O-H]-	530-57-4	7092758.777
362	Indole-3-carboxylic acid	162.05	2.19	C9H7NO2	[M+H]+	771-50-6	7068307.992
363	6-aldehydoisoophiopogonone a	372.15	1.18	C19H14O7	[M+NH4]+	112500-90-0	7060165.901
364	Scolymoside	595.17	4.81	C27H30O15	[M+H]+	25694-72-8	7036644.887
365	Ricinine	182.09	1.38	C8H8N2O2	[M+NH4]+	524-40-3	6992366.295
366	Syringaldehyde	181.05	4.87	C9H10O4	[M-H]-	134-96-3	6986382.535
367	Forsythoside b	801.25	3.97	C34H44O19	[M+HCOO]-	81525-13-5	6926514.625
368	Quercitrin	449.10	4.75	C21H20O11	[M+H]+	522-12-3	6906997.118
369	Isoimperatorin	251.03	4.79	C16H14O4	[M-H2O-H]-	482-45-1	6899553.102
370	Ginsenoside rg5	749.48	6.25	C42H70O12	[M-H2O+H]+	186763-78-0	6894974.927
371	2-methylbenzoic acid	135.05	5.59	C8H8O2	[M-H]-	118-90-1	6890949.813
372	4-demethyl-	383.11	6.64	C21H20O7	[M-H]-	3590-93-0	6876422.522
373	4'-demethylepipodophyllotoxin	799.22	4.98	C21H20O8	[2M-H]-	6559-91-7	6849694.488
374	Bergamottin	356.17	4.75	C21H22O4	[M+NH4]+	7380-40-7	6847572.318
375	Dihydrosanguinarine	667.21	5.81	C20H15NO4	[2M+H]+	3606-45-9	6831089.168
376	Camptothecin	349.18	10.68	C20H16N2O4	[M+H]+	7689-03-4	6808765.858

377	Coniferaldehyde	179.07	5.22	C10H10O3	[M+H]+	458-36-6	6768441.76
378	Scandoside	389.11	0.93	C16H22O11	[M-H]-	18842-99-4	6766213.477
379	Guanidinosuccinic acid	176.05	1.19	C5H9N3O4	[M+H]+	6133-30-8	6716942.578
380	Savinin	413.15	4.02	C20H16O6	[M+HCO3]-	493-95-8	6704701.32
381	Narcissin	623.16	4.75	C28H32O16	[M-H]-	604-80-8	6445069.954
382	Kurarinol	457.22	3.70	C26H32O7	[M+H]+	855746-98-4	6400451.31
383	Citral	213.11	1.46	C10H16O	[M+HCO3]-	141-27-5	6347944.082
384	Sugiol	299.06	7.13	C20H28O2	[M-H]-	511-05-7	6339923.93
385	Delphinidin 3,5-diglucoside	611.14	4.61	C27H31O17+	[M-NH3+H]+	17670-06-3	6176711.922
386	Quercetin 3-(6'-malonyl-glucoside)	551.10	4.87	C24H22O15	[M+H]+	96862-01-0	5958985.16
387	1-methoxy-4-(2-propenyl)benzene	166.12	4.03	C10H12O	[M+NH4]+	140-67-0	5885314.896
388	Cucurbitacin o	501.32	10.17	C30H46O7	[M-H2O+H]+	25383-23-7	5802037.751
389	Angulatin a	661.27	6.47	C34H46O13	[M-H]-	139979-81-0	5715041.768
390	Quercetin 3,3-bissulfate	462.85	0.71	C15H10O13S2	[M+H]+	108909-05-3	5676224.238
391	2,4-diaminobutyric acid	117.06	0.85	C4H10N2O2	[M-H]-	1758-80-1	5623316.99
392	Artemisitene	279.12	2.12	C15H20O5	[M-H]-	101020-89-7	5606098.127
393	Delphinidin	287.03	4.88	[C15H11O7]+	[M-NH3+H]+	528-53-0	5524647.003
394	Iridin	521.22	4.72	C24H26O13	[M-H]-	491-74-7	5438993.18

395	2'-o-methylisoliquiritigenin	315.09	6.71	C16H14O4	[M+HCOO]-	112408-67-0	5416934.591
396	Dihydrochelirubine	402.21	9.67	C21H17NO5	[M+K]+	28342-26-9	5406854.995
397	Ginsenoside f2	829.50	7.10	C42H72O13	[M+HCOO]-	62025-49-4	5398033.143
398	Medicagenic acid	501.32	6.76	C30H46O6	[M-H]-	599-07-5	5269069.848
399	Ginsenoside rh3	587.43	6.43	C36H60O7	[M-H2O+H]+	166040-90-0	5246835.534
400	Perilla ketone	167.07	4.75	C10H14O2	[M+H]+	553-84-4	5215084.435
401	Hirsuteine	427.19	4.36	C22H26N2O3	[M+HCO3]-	35467-43-7	5133112.149
402	Piceol	197.05	1.41	C8H8O2	[M+HCO3]-	99-93-4	5131148.422
403	2,4,5-trimethoxybenzaldehyde	195.07	4.47	C10H12O4	[M-H]-	4460-86-0	5070077.816
404	Isoliquiritin	417.12	5.02	C21H22O9	[M-H]-	5041-81-6	5048145.522
405	Isofraxidin	221.05	3.93	C11H10O5	[M-H]-	486-21-5	5032213.464
406	Apiin	563.15	5.06	C26H28O14	[M-H]-	26544-34-3	4966391.716
407	Pelargonin	579.18	4.70	C27H31O15+	[M-OH+H]+	17334-58-6	4945670.008
408	Carpachromene	335.09	8.30	C20H16O5	[M-H]-	57498-96-1	4876746.983
409	3,4-di-o-caffeoylquinic acid	517.13	1.80	C25H24O12	[M+H]+	14534-61-3	4800450.172
410	Sanggenon c	707.19	6.78	C40H36O12	[M-H]-	80651-76-9	4732258.197
411	Artemisinin	327.15	4.79	C15H22O5	[M+HCOO]-	63968-64-9	4723999.311
412	Chelerythrine	329.11	5.25	C21H18NO4+	[M-H2O-H]-	34316-15-9	4650482.361

413	3beta,4beta,5-trimethoxy-4-hydroxy-(6_7)-2,2-dimethylpyranoflavan	397.17	8.24	C23H26O6	[M-H]-	462109-98-4	4612491.236
414	Magnolol	265.12	8.23	C18H18O2	[M-H]-	528-43-8	4464015.734
415	Rosiridin	331.19	7.92	C16H28O7	[M-H]-	100462-37-1	2038613.284
416	Xanthohumol	353.14	9.99	C21H22O5	[M-H]-	6754-58-1	2020434.58
417	Tanshinone iia	317.11	0.71	C19H18O3	[M+Na]+	568-72-9	1974371.308
418	Naringin	581.18	5.14	C27H32O14	[M+H]+	10236-47-2	1966740.934
419	Dehydroabietic acid	323.19	4.89	C20H28O2	[M+Na]+	1740-19-8	1962350.23
